# Coping in youth living with chronic pain: A systematic review of qualitative evidence

**DOI:** 10.1080/24740527.2025.2455494

**Published:** 2025-02-25

**Authors:** Roberta L. Woodgate, Ashley Bell, Julianna Petrasko, Christine J. Neilson, Olabisi Ayeni

**Affiliations:** a College of Nursing, Rady Faculty of Health Sciences, University of Manitoba, Winnipeg, Manitoba, Canada; b Neil John Maclean Health Sciences Library, University of Manitoba, Winnipeg, Manitoba, Canada

**Keywords:** chronic pain, youth, coping, systematic review, qualitative, youth experiences, health care system

## Abstract

**Background:**

Chronic pain is progressively receiving attention as a universal public health priority. It is anticipated that there will be an increase in the prevalence of chronic pain in the coming years, particularly among youth. Chronic pain can be stressful and have a significant impact on young people and their family.

**Aims:**

The aim of this systematic review was to synthesize the best available qualitative evidence on the coping experiences of youth living with chronic pain and to note whether there were any differences in their coping experiences.

**Methods:**

A multi-database search was conducted including child development and adolescent studies. CINAHL, MEDLINE, PsycINFO, Embase, and Scopus were searched for eligible English-language articles from inception to December 2023. Out of 1625 article titles and abstracts screened for eligibility, 280 articles underwent full-text screening, with 20 ultimately meeting all inclusion criteria. We conducted a thematic analysis of data extracted from the 20 reviewed articles.

**Results:**

We arrived at two synthesized findings. *A Different Way of Being* considers the experience of being a youth with chronic pain. *Learning to Get By* looks at the coping strategies youth use to manage their chronic pain and involved youth using *self-directed strategies*, as well as relying on *external supports*.

**Conclusions:**

It is apparent from these synthesized findings that youths’ lives have been significantly impacted by chronic pain. Findings from this study can be used to support the care and well-being of youth living with chronic pain.

## RÉSUMÉ

**Contexte :** La douleur chronique retient de plus en plus l’attention en tant que priorité de santé publique mondiale. On prévoit une augmentation de la prévalence de la douleur chronique dans les années à venir, particulièrement chez les jeunes. La douleur chronique peut être stressante et avoir un effet important sur les jeunes et leur famille.

**Buts :** Le but de cette revue systématique était de synthétiser les meilleures données probantes qualitatives disponibles sur les expériences d'adaptation des jeunes vivant avec de la douleur chronique et d’examiner les différences dans leurs expériences d'adaptation.

**Méthodes :** Une recherche a été menée dans des bases de données multiples, pour y repérer les études européennes sur le développement de l'enfant et de l'adolescent. Des recherches ont été effectuées dans CINAHL, MEDLINE, PsycINFO, Embase et Scopus pour rechercher les articles en langue anglaise admissibles depuis leur création jusqu'en décembre 2023. Sur 1 625 titres d'articles et résumés dont l'admissibilité a été vérifiée, 280 articles ont fait l'objet d'une sélection en texte intégral, 20 d'entre eux répondant finalement à tous les critéres d'inclusion. Nous avons effectué une analyse thématique des données extraites des 20 articles examinés.

**Résultats :** Nous avons dégagé deux résultats synthétisés. *A Different Way of Being* aborde l’expérience d'un jeune vivant avec la douleur chronique. *Learning to Get By* examine les strat'gies d'adaptation que les jeunes utilisent pour gérer leur douleur chronique en mettant en lumière l’utilisation de stratégies autogérées, ainsi que le recours à un soutien externe.

**Conclusions :** Ces résultats synthétisés montrent que la vie des jeunes est considérablement affectée par la douleur chronique. Les résultats de cette étude peuvent être utilisés pour soutenir les soins et le bien-être des jeunes vivant avec de la douleur chronique.

## Introduction

Chronic pain is progressively garnering attention as a universal public health priority,^1,2^ having been included in the *International Classification of Disease and Related Health Problems*, a global classification systems of diseases and health related problems, in 2021.^3,4^ Defined as persistent or recurring pain that lasts more than 3 months, chronic pain is an unpleasant sensory experience that may or may not be associated with a physical injury.^3^ The experience of pain is unique to the individual and can be affected by biological, psychological, and social factors.^2,5^ Current research predicts an upsurge in the prevalence of chronic pain, especially among youth (i.e., young people aged 12–21 years).^6–9^

**Figure 1.**
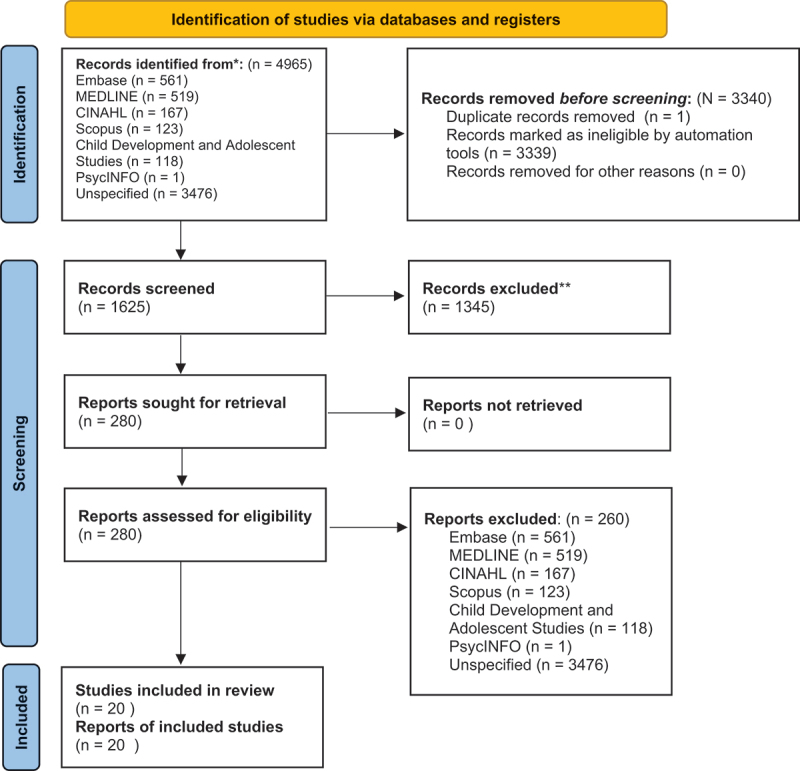
PRISMA flow diagram. *Consider, if feasible to do so, reporting the number of records identified from each database or register searched (rather than the total number across all databases/registers). **If automation tools were used, indicate how many records were excluded by a human and how many were excluded by automation tools.

**Figure 2.**
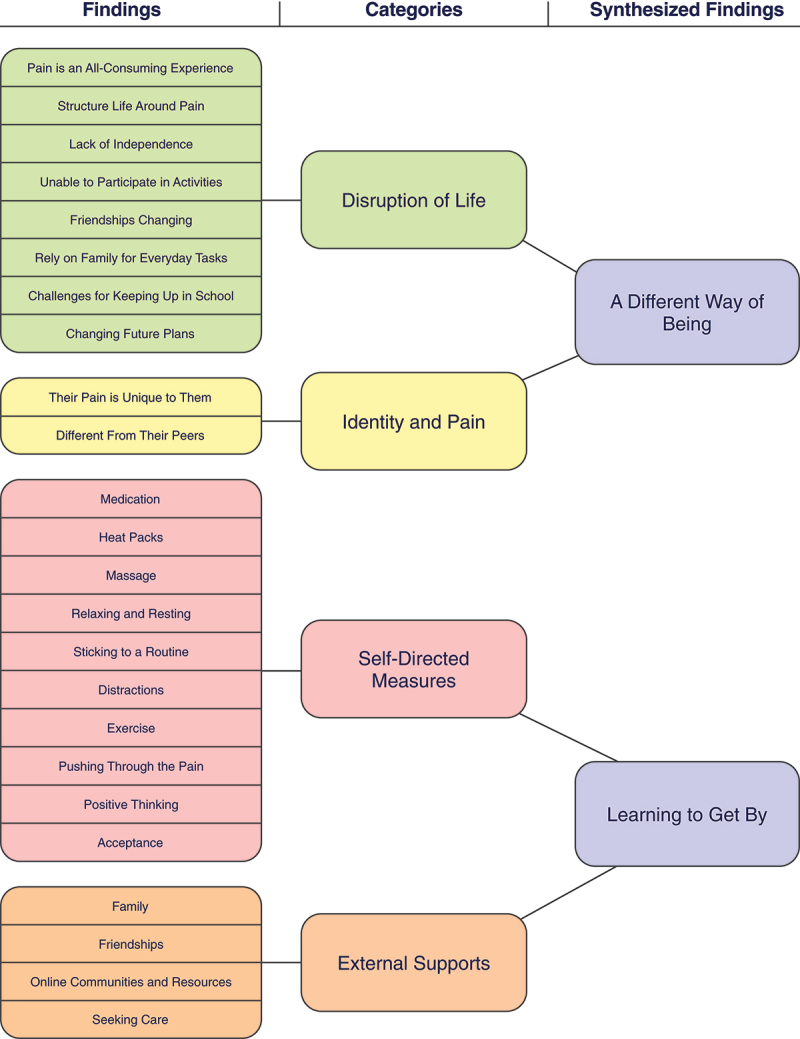
Summary of synthesized findings.

Chronic pain affects an estimated 20% of people worldwide,^3,10–12^ with a similar rate of 20.8% of youth globally experiencing chronic pain.^13^ These rates can vary, with a higher prevalence of chronic pain seen in youth who are girls,^13–17^ have a lower socioeconomic status,^14^ are a racial minority,^18^ and are older.^18^ Many youth report chronic headache, abdominal, back, and musculoskeletal pain,^5,13,14,16,17,19^ and among youth who experience chronic pain, 20.6% note experiencing pain in at least two body sites.^16^

Chronic pain can be stressful, and its impact is pervasive. An undiagnosed chronic pain condition, alongside diagnostic uncertainty, can be stressful for youth and families.^20^ Even with a diagnosis, youth can experience confusion and uncertainty about their condition and how it will impact their life.^20^ Youth living with chronic pain can experience functional impairment and worse general well-being.^15,18^ They may also experience increased school absenteeism,^15,21^ anxiety,^7,14,22,23^ depression,^7,14,22–25^ and loneliness,^26^ as well as lower quality of life^15,18^ and self-esteem.^14^ Chronic pain can impact the development of youth and can contribute to mental health challenges and substance use disorders into their adulthood.^2,7,9,23,27,28^ Their pain experience often has a significant impact on their family members as well.^2,5^

Approximately one out of three youth with chronic pain struggle to cope with their pain.^25^ Coping, defined within the context of youth well-being, is the “conscious and volitional efforts to regulate emotion, cognition, behaviour, physiology, and the environment in response to stressful events or circumstances.”^29(p89)^ Youth can utilize a range and mix of different approaches to help them cope with their chronic pain, such as distraction, deep breathing exercises, and talking with others about their pain.^30,31^ Youth with chronic pain who are able to utilize effective coping strategies have improved quality of life.^31,32^ The effectiveness of such coping strategies also corresponds to youths’ pain levels, with coping strategies being more effective for lower levels of chronic pain.^31^

Little attention has been given to how youth cope with chronic pain, despite the long-term risks for social and economic disparities,^33^ and their persisting challenges into adulthood, such as higher levels of depression and anxiety.^7–9^ Reviews on coping with chronic pain among youth are often quantitative.^34^ Though such reviews have contributed to the knowledge of coping with chronic pain, they lack the ability to explore the unique meanings behind chronic pain experiences. Existing qualitative syntheses of chronic pain in youth have explored the experiences of youths’ fluctuating pain in musculoskeletal disorders,^35^ examined the experiences coping with chronic pain among youth who had a history of psychological trauma,^36^ looked at the impact of chronic pain on youths’ school functioning,^21^ and the experiences of youth living with nonclinical chronic pain.^37^ At present, no qualitative systematic review has captured the experiences of youth coping with chronic pain across diagnosed and undiagnosed conditions.

Considering this limitation, a synthesis of the literature focusing on the coping experiences of youth living with chronic pain will be useful to arrive at an increased understanding of their experiences, which can then be utilized to help us support youths’ health and well-being.^25^ Therefore, the aim of this systematic review was to synthesize the best available qualitative evidence on the lived experiences of youth living with chronic pain, with or without a formal medical diagnosis, with attention to how they cope. Specifically, the following questions were addressed:

1. What meanings do youth assign to living with chronic pain?

2. How do youth cope with their chronic pain?

## Methods

This review is reported in accordance with the enhancing transparency in reporting the synthesis of qualitative research (ENTREQ) statement, which consists of 21 items for assessing the searching and selecting of qualitative research, quality appraisal, and methods for synthesizing qualitative findings (see supplemental material).^38^

A professional librarian (C.J.N.) designed a draft search strategy to identify relevant literature in Ovid Medline with input from members of the research team. A second librarian peer reviewed the search using the 2015 PRESS guideline and checklist.^39^ The finalized search was translated and run in each of the following databases: Child Development and Adolescent Studies (EBSCO); CINAHL with Fulltext (EBSCO); Ovid MEDLINE(R) and Epub Ahead of Print, In-Process, In-Data-Review & Other Non-Indexed Citations and Daily; Embase (Ovid); APA PsycInfo (Ovid); and Scopus (Elsevier). A multi-database search was conducted in Sociological Abstracts and Social Services Abstracts via the ProQuest platform. Searches were last updated in the databases listed above on December 4, 2023.

The search incorporated a modified version of DeJean and colleagues’ qualitative research search filter to focus search results on qualitative and mixed methods research.^40^ An additional filter was used in Scopus to exclude Medline and Embase records from the Scopus search.^41^ No other limits or filters were used. Duplicate records were identified and removed from the initial result set using Endnote.^42^ Records identified when the search was updated were deduplicated by Covidence software.^43^ Complete search histories for each database are available in the University of Manitoba institutional repository.^44^

The criteria for studies to be included were as follows: (1) it must be a qualitative study using any study method. Mixed method studies were included if the qualitative findings were included separately; (2) qualitative findings must include reports directly from youth themselves, and not through a proxy, such as a caregiver or parent; (3) strategies or management approaches for coping with chronic pain must be reported in the findings; (4) youth reporting must be between 12 and 21 years old (mean age of >12 or <21 if a broader age range sampled) and must live with chronic pain (defined as consistent or recurrent pain lasting longer than 3 months with or without a clear physical pathology or diagnosis); and (5) it must be published in a peer-reviewed English language journal. It should be noted that though youth is best understood as a period of transition from the dependence of childhood to adulthood’s independence, the age range does vary depending on the source.^45^ For the purposes of this review, the age range of 12 to 21 years was selected in recognition that chronic pain becomes prominent for many youth during adolescence^46^ and that around 21 years is when youth transition to adult-based care.^47^

Studies were excluded if (1) the complete study was not available, (2) the paper was a meta-analysis or meta-synthesis, (3) it was a different topic area, (4) it was not original research, (5) no qualitative findings were reported, (6) the findings were not reported by youth, (7) youth did not belong to the correct age group, (8) the paper reported on acute pain only, or (9) there were no findings on coping strategies.

The initial search of the databases identified 4965 articles (see Figure 1), which were then imported into Covidence. After duplicates were removed, 1625 studies remained. The article abstracts were then assessed by two reviewers (J.P. and O.A.), and articles were removed if they did not fit the inclusion criteria. This left 280 articles for further screening. The second screening consisted of three reviewers (R.L.W., J.P., and O.A.) reading the full article and including articles based on the inclusion criteria. A total of 30 studies were included. After further discussion among the authors (R.L.W., A.B., J.P., and O.A.), ten articles were dropped from the review because they did not meet the inclusion criteria, leaving 20 articles at this point.

### Quality Assessment

The Mixed Methods Appraisal Tool (MMAT), which provides guidance for appraising the methodological quality of qualitative, quantitative randomized controlled, quantitative nonrandomized, quantitative descriptive, and mixed method studies, was utilized for the quality assessment.^48^ The methodological quality assessment was done by two reviewers (R.L.W. and J.P.) independently for each paper included at this stage. A quality score was given to each study using the Mixed Methods Appraisal Tool. For each section, either a value of 1 for “yes” responses or 0 for “no” responses were given. Any disagreements from the quality assessment were reviewed and discussed by four reviewers (R.L.W., A.B., J.P., and O.A.) until a consensus was agreed upon. At this point any article that was found to not meet a high methodological standard was excluded from the study. All 20 articles that met the inclusion criteria also met the quality assessment standard, and, as such, all were included in this systematic review for data extraction.

### Data Extraction

Three reviewers (R.L.W., J.P., and O.A.) extracted the data relevant to the research aim and questions from the 20 studies (see Table 1). The extracted data table included information pertaining to authors’ names, year published, country the study was conducted in, study design, purpose of the study, recruitment setting, age of the participating youth, data collection method, number of participating youth, participants’ type of chronic pain, main findings of the study, and limitations identified. Any disagreements during the extraction were discussed among three reviewers (R.L.W., A.B., and J.P.) until an agreement was reached.

**Table 1.** Characteristics of studies in the systematic review.

Study (country)	Design, purpose	Recruitment setting, youth age	Data collection method	Number of youth (male/female)	Type of chronic pain	Main findings	Limitations
Alsaggaf and Coyne^63^ (Saudi Arabia)	Multiple case study Report on the experience of chronic pain, its impact on participation in everyday life, and strategies to manage chronic pain	Large tertiary hospital 12–16 years Mean age of 14.8	Semistructured face-to-face and telephone interviews	*N* = 10 (5 males, 5 females) Additional participants included parents (*N* = 10) and school personnel (*N* = 20)	• Sickle cell anemia • Celiac disease Systemic lupus erythematous • Migraine • Osteogenesis imperfecta • Chronic sinusitis • Headache • Muscular dystrophy • Juvenile rheumatoid arthritis • Ulcerative colitis • Crohn’s disease	• Themes: (1) Experiencing chronic pain: “Like a big rock on me. … I cannot bear it.” (2) Impact of pain on quality of life: “Pain ruins everything.” (3) Everyday strategies to manage chronic pain: “I don’t let the pain disrupt my day.” • Coping strategies used: medications, herbal remedies, massage, hot/cold, drinking water, eating healthy, positive thinking, relaxation, distraction, exercise, spirituality, family support, avoiding triggers	• Only involved youth with secondary chronic pain • Potential bias due to recruitment from tertiary hospitals and schools in specific regions • Phone interviews may have obscured nonverbal clues during COVID-19. • Differences in male and female experiences were not highlighted. • Youth ethnicity/race not reported
Carter et al.^53^ (United Kingdom)	Qualitative, participant inquiry study Explore the way in which the experience of chronic pain impacts the lives of young people	Children’s hospital 13–19 years	Post-it® pyramids (identifying and prioritizing key words depicting pain experiences), peer interviews, and a focus group interview	*N* = 5 (gender not specified)	Abdominal pain • Headaches • Bone pain • Back pain	• Themes: (1) “No one’s pain’s the same, it’s always there.” (2) “Getting on with it.” (3) “It’s hard ’cos … but we’re keeping with the dream.” (4) “It depends … *some* are okay.” • Coping strategies used: relaxing, talking with others, family support. • Young people experienced significant disruption in their lives because of their pain. • Professionals need to develop insight and strategies into the challenges young people with chronic pain face daily.	• Small sample size • All participants were receiving tertiary level management of their pain and thus may be unrepresentative of young people receiving secondary level or primary care management of their chronic pain. • Youth ethnicity/race not reported
Castle et al.^51^ (Australia)	Phenomenological study Explore the experience and impact of chronic pain on the lives of adolescents and young adults with cerebral palsy	Outpatient setting in a children’s hospital 14–24 years Mean age of 17.6	In-depth interviews	*N* = 6 (4 males, 2 females)	• Hip pain • Back pain • Limb pain • Bladder pain • Hand, wrist, and shoulder pain • All participants had cerebral palsy.	• Themes: (1) The experience of chronic pain. (2) Making sense of the pain. (3) Doing anything to get rid of the pain. (4) Fighting the pain. (5) Enlisting help. (6) Being in pain. (7) Looking ahead. • Coping strategies used: relaxation, medication, splints, family support	• Differences in male and female experiences were not highlighted. • There was no discussion on how the type of cerebral palsy may have shaped the participants’ experiences. • Youth ethnicity/race not reported
Forgeron and McGrath^57^ (Canada)	Qualitative approach Explore the self-identified needs of adolescents living with chronic pain	Tertiary care pediatric chronic pain clinic 13–17 years	Focus group	*N* = 6 (1 male, 5 females)	• Pelvic pain • Shoulder pain • Neck pain • Hand pain • Foot pain • Knee pain • Stomach pain	Themes: (1) Struggling to be normal. (2) Dealing with the pain. • Coping strategies used: medications, distraction, support from friends with chronic pain • School was the greatest source of stress. • Youth were unsure what skills they needed to transition into adult care and asserted that they were not ready to do so.	Data were only collected in one session, which could have limited the number of needs uncovered. • Differences in male and female experiences were not highlighted. • All participants were Caucasian and middle-class.
Forgeron et al.^61^ (Canada)	Interpretive phenomenology To understand the challenges adolescents with pain face regarding their friendships	Tertiary pain clinic and community-wide advertisement 14–18 years	Individual interviews	*N* = 8 Additional participants included youth without chronic pain (*N* = 8) (total *N* = 16: 2 males, 14 females)	• Widespread chronic pain • Headaches • Musculoskeletal pain	Themes: (1) Rethinking the self with pain. (2) Rethinking friendships. • Coping strategies used: support from close friends, draw on inner strength	• With only two males participating in the study, it was not possible to analyze sex differences. • Youth ethnicity/race not reported
Ghio et al.^62^ (United Kingdom)	Qualitative cross-sectional study Examine adolescents’ goals when coping with pain from juvenile idiopathic arthritis and map these goals to the cognitive and emotional profiles of both the adolescent and their parent	Hospital through ongoing Childhood Arthritis Prospective Study 11–16 years	Two-part interview for adolescents, qualitative survey for parents	*N* = 17 (7 males, 10 females) Additional participants included parents of youth (*N* = 17)	• Juvenile idiopathic arthritis	• Coping strategies used: • (1) Physical activity: (a) resting and relaxing, (b) staying still, (c) physical activity, (d) get on with it (normalize their pain). • (2) Pain disclosure: (a) not telling at all, (b) telling mum but not school or friends, (c) telling anyone. • (3) Treatment: (a) seeking support from health professionals, (b) taking medication.	• The adolescents’ pain representations may have been influenced by the parental pain beliefs, which may not accurately reflect the individual experiences of the adolescents. • Adolescent ethnicity/race not reported
Gremillion et al.^55^ (United States)	Qualitative approach with interpretive phenomenological analysis Understand the challenges experienced by adolescents with co-occurring chronic pain and obesity, with a specific focus on physical activity	Multidisciplinary pediatric pain and headache clinic 13–17 years Mean age of 15.5	Semistructured phone interviews	*N* = 13 (2 males, 11 females)	• Headaches • Generalized pain • Abdominal pain • Back pain • Lower extremity pain • All participants had obesity.	• Themes: (1) Impact of chronic pain on relationships. (2) Impact of pain on self-perception. (3) Using food to cope with pain. (4) Perceived relationship between pain and weight after onset of pain. (5) Attitudes toward physical activity. (6) Barriers to physical activity. (7) Supports to physical activity. • Coping strategies used: avoid triggering foods, support from friends/family with chronic pain	• Phone interviews may have obscured nonverbal clues. • Pain onset was not accounted for, despite onset ranging from 4 months to over 7 years. • Differences in male and female experiences were not highlighted. • Diverse ethnicity/race of youth but not considered in analysis
Hurley-Wallace et al.^69^ (United Kingdom)	Qualitative design Explore young people’s experiences of searching for information about chronic pain using the internet	Community, advertised online and through relevant charities and a sixth form college 16–24 years Mean age of 21.1	Online, semistructured individual interviews	*N* = 24 (1 male, 21 females, 1 gender variant)	• Primary chronic pain • Posttraumatic pain • Headache and orofacial pain • Visceral pain • Musculoskeletal pain • Diagnoses included: • Joint hypermobility syndrome • Chronic headache • Migraine • Fibromyalgia • Ehlers-Danlos syndrome • Endometriosis • Rheumatoid arthritis	• Themes: (1) Trustworthy information or experiences? (2) Diagnostic labels in a digital world. (3) The online chronic pain community. (4) A mind and body approach to self-management. • Coping strategies used: online mindfulness, meditation, and yoga resources • Youth trusted advice from others in their online community. Having a diagnostic label helped them find relevant management strategies and support networks online.	• The sample did not represent young people with cancer-related or neuropathic pain and disproportionally represented participants who are female. • Sample population was largely White, and no analysis of ethnicity.
Jones et al.^55^ (United Kingdom)	Qualitative design Explore how adolescents make sense of their experience of chronic pain in the context of their social development	National Health Service tertiary pain service 12–22 years Mean age of 15.7	Telephone interviews, monthly diaries	*N* = 9 (1 male, 8 females)	• Chronic coccyx pain • Eczema • Chronic neuropathic pain of the skin • Hypermobility • Persistent localized leg pain • Chronic musculoskeletal pain • Chronic ankle pain • Chronic back pain • Chronic abdominal pain	• Overarching theme: “tug of war: push and pull” (developmental tension related to pain and the cumulative impact it had over time). This comprised subthemes: (1) The shifting sands of peer relationships. (2) Restricted choices. • Coping strategies used: isolating themselves from peers, distraction, prioritizing time with friends	• Participants were primarily girls, all identified as White, and all spoke English as a first language. • Three participants did not complete the diary portion. • Wide age range of participants (12–22) led to notable differences in their developmental stage, relevant concerns, and experiences. This made it challenging to understand specific concerns at each developmental stage.
Killackey et al.^58^ (Canada)	Qualitative descriptive design Describe the impact of the COVID-19 pandemic on the experiences of Canadian families of youth living with chronic pain	Pediatric chronic pain clinics across Canada, national pain and children’s health organizations, social media Mean age of 16.5 years	Semistructured interviews	*N* = 19 (2 males, 17 females) Additional participants included parents (*N* = 14) and siblings (*N* = 11)	• Specific chronic pain types and/or condition(s) not reported	• Themes: (1) Absorbing and shifting: the toll of the pandemic on the family system including loss of coping mechanisms. (2) Social ambiguity and abandonment. (3) Building community resilience: familial adaptation to the pandemic. • Coping strategies used: support from siblings and parents, meditating, exercise • Youth expressed the effects of the pandemic on pain management. Coping mechanisms were limited, with a lack of distraction opportunities, and accessing quite time in the home was difficult with the presence of family members and pets.	• Youth were predominantly White, with parents who were educated and had higher socioeconomic status. • Interviews were conducted at only one time point. As such, they reflect a certain phase of the pandemic, which was prior to widespread vaccination in Canada.
Mahon and Reynolds^71^ (Canada)	Interpretative description Explore the lived experiences of adolescents with a diagnosis of primary chronic pain to understand their perception of onset and the impact of their condition	Chronic pain clinic 13–19 years	Virtual semistructured interviews	*N* = 15 (4 males, 11 females)	• Primary chronic pain	• Themes: (1) Psychological: (a) diagnostic uncertainty and uncertainty about the future, (b) depression and anxiety, (c) professionals’ skepticism, (d) importance of peer support, (e) importance of parental support. (2) Physiological: (a) effect on activities of daily living, (b) sleep, (c) exercise, sports, recreation. (3) Systematic: (a) school, (b) lack of resources, (c) delays in referrals. • Coping strategies used: support from parents, support from friends • Youth perceived that health care professionals did not believe their pain and its intensity.	• Youth ethnicity/race and culture not reported. • Gender differences in pain perception were not analyzed. • Information on previous hospitalization was not gathered. • Limited to a single chronic pain clinic, and findings may be different at other clinics.
McKinnon et al.^66^ (Australia)	Convergent parallel mixed methods design, including quantitative prospective cross-sectional and qualitative interviews Explore the lived experience of chronic pain and dyskinesia in children and adolescents with cerebral palsy	Tertiary hospitals 9–16 years (qualitative phase) Mean age of 13.3	Semistructured interviews (qualitative phase)	*N* = 8 (6 males, 2 females) for the qualitative phase	• Abdominal pain • Back pain • Limb pain • Face/temple/jaw pain • Head pain • Throat/neck pain • Chest pain • Groin/pubic pain • All participants had cerebral palsy.	• Themes: (1) Lives embedded with dyskinesia. (2) Real-world challenges of chronic pain. (3) Still learning strategies to manage their pain and dyskinesia. • Coping strategies used: push through the pain, stretching, massage, distraction, hot/cold, positioning, medications, rest, controlled breathing, seeking comfort from family	• Homogenous sample used for qualitative interviews. Race/ethnicity of participants not reported • There may have been differences in pain experiences between younger childhood (9–12 years) and adolescence (>12 years), and across participants with differing levels of dyskinesia severity.
Meldrum et al.^61^ (United States)	Mixed methods study with quantitative and qualitative procedures Examine the impact of pain-associated functioning limitations on children’s lives and the strategies they develop to try to continue functioning	Tertiary chronic pain clinic 10–18 years (qualitative phase) Mean age of 14.7	Semistructured interviews (qualitative phase)	*N* = 45 (13 males, 32 females)	• Headache • Myofascial pain • Functional neurovisceral pain disorder • Complex regional pain syndrome • Fibromyalgia	• Three functioning patterns of youth were identified: (1) Adaptative. (2) Passive. (3) Stressed. • Coping strategies used: medications, rest/sleep, distraction, changing eating or other habits, crying, focusing on the pain • Three recurrent strategies were identified: (1) Getting on with things. (2) Planning ahead. (3) Body awareness.	• Ethnic, cultural, and gender differences in pain perceptions were not reported.
Nkhoma et al.^62^ (Malawi)	Qualitative design Explore stakeholders’ perspectives and experiences of pain self-management for adolescent living with HIV and chronic pain	HIV primary care clinic 10–17 years Mean age of 14.7	In-depth interviews	*N* = 21 (11 males, 10 females) Additional participants included family caregivers of these youth and health care providers in HIV clinics	• All participants had chronic pain with co-occurring HIV.	• Themes: (1) Experiencing physical, psychological, and spiritual pain. (2) Self-management approaches for pain and symptoms. (3) Current pain strategies among adolescents. • Coping strategies used: medication (Western and traditional); walking; exercise/jogging; reading magazines; drinking cold water; washing the painful body part with cold water; spending time with friends; support from friends, parents, and health care providers; watching TV	• Recruitment relied on staff being aware of youths’ pain, but their awareness may have been low. • There may have been selection bias. Youth with severe pain who dropped out of care may not have been approached for recruitment.
Parsons et al.^68^ (United Kingdom)	Qualitative design Explore how adolescents experience, understand, and perceive positive growth while living with chronic pain	Previous study that recruited from tertiary care pediatric-specific pain clinics and social media 11–24 years Mean age of 17.8	Diary entries, semistructured interviews	*N* = 24 (1 male, 21 females, 1 trans male, 1 non-binary)	• Complex regional pain syndrome • Fibromyalgia • Ehlers-Danlos syndrome • Psoriatic arthritis • Chronic migraine • Crohn’s disease • General chronic pain • Craniopharyngioma Scoliosis Bone marrow edema Ankylosing spondylitis • Joint hypermobility syndrome	• Themes: (1) Appreciating the moment. (2) Becoming a better version of myself. • Coping strategies used: distraction through hobbies/skills (crochet, knitting, learning new languages, and art)	• Follow-up interviews were not conducted with the full sample of youth who participated in the diary entry portion. • Most of the youth were White and female.
Root and Nosek^58^ (United States)	Exploratory design Provide insight into the language that college students use to describe their experiences with chronic pain, challenges they face, and coping strategies they use	A liberal arts college Mean age of 20	Qualitative writing exercise	*N* = 11 (1 male, 10 females)	• Neck/spine pain • Headaches/migraines • Stomach/digestive pain • Endometriosis • Fibromyalgia • Tendonitis • Joint pain/arthritis	• Challenges living with chronic pain: (1) Lack of permanent solutions. (2) Impact on mental health. (3) Need for social support. (4) Academic challenges. • Coping strategies used: exercise, support from others (not specified who), self-care (rest, sleep, breaks), medications, alternative medicine, mental health therapy, negative thinking (anger, catastrophizing, ruminating, shutting down, jealousy), self-isolation, avoidance, not advocating for oneself • Youth were more likely to use negatively valanced words than positively valanced words to describe their pain.	• Participants were predominantly female and middle-class or higher, all White, and all from the same college.
Shaygan and Jaberi^65^ (Iran)	Mixed methods design Assess a smartphone‐based pain management application regarding the feasibility, adherence, participant satisfaction, and effectiveness on pain intensity and quality of life in adolescents with chronic pain	From a baseline sample of a previous study 12–19 years Mean age of 13.7	Semistructured interviews (qualitative phase)	*N* = 14 (5 males, 9 females) for the qualitative phase	• Chronic pain, specific type(s) and/or condition(s) not reported	• Categories of pain management strategies: (1) Physical management (analgesics, topical methods such as heat, traditional/herbal medicine, and topical medications such as ointments). (2) Psychological management (distraction, mental avoidance/not thinking about the pain, resilience, optimism, spirituality). (3) Interpersonal resources (support from family, peers, school officials). • The smartphone‐based pain management program had a positive effect on pain intensity and quality of life.	• Gender and age were not considered in the analysis of the interviews. • Youth ethnicity/race not reported
Skogvold and Magnussen^67^ (Norway)	Qualitative design Explore the strategies youth use to cope with chronic tension-type headaches	Pediatric neurologists, physiotherapists, and public health nurses invited eligible youth 14–17 years	In-depth interviews	*N* = 17 (6 males, 11 females)	• Chronic tension-type headache	• Main categories of coping strategies: (1) Structure and routines in daily life. (2) Low-level activity and walking are best. (3) Rest, relaxation, and withdrawal. (4) Therapy—expectations and disappointments. (5) thoughts on use of medication in connection with headaches.	• The quality of the interviews with younger participants was considered somewhat low because the younger participants were not as willing to share their experiences. • It was noted that the interviewer may have misinterpreted the participants, which could impact data validity. • Youth ethnicity/race not reported
Stinson et al.^67^ (Canada)	Descriptive exploratory qualitative design Conduct a user-centered needs assessment to inform the development of an integrated web- and smartphone-based self-management program for adolescents with chronic pain, called iCanCope with Pain^TM^	Pediatric tertiary care chronic pain clinics 14–18 years	Focus groups, individual interviews	*N* = 22 (5 males, 17 females)	• Headache • Abdominal pain • Widespread pain • Lower back pain • Neuropathic pain • Facial pain • Other chronic pain, not included in the above	• Themes: (1) Pain impact. (2) Barriers to care. (3) Pain management strategies. (4) Transition to adult care. • Coping strategies used: support from friends, physical therapies, distraction, hobbies, extracurricular activities	• Study sample was from two urban, primarily English-speaking centers in southwestern Ontario in Canada. • Youth ethnicity/race not reported
Szwimer et al.^59^ (Canada)	Interpretive phenomenological design Inquiry into the experiences of female youth living with chronic pain to enhance our understanding of how chronic pain impacts their personal lives	Pediatric chronic pain management outpatient clinic 14–17 years	Individual interviews	*N* = 8 (all females)	• Chronic back pain • Abdominal pain • Hand pain • Pelvic pain • Orofacial pain • Widespread pain • Fibromyalgia • Arthritis	• Factors facilitating one’s ability to live well: (1) Creating a safe internal space. (2) Accepting the incurability of chronic pain. (3) Envisioning a fulfilling future. • Factors hindering one’s ability to live well: (1) Facing the unpredictability of chronic pain. (2) Facing the uncertainty of chronic pain. • Coping strategies used: drawing, listening to music, singing, reading, photography, walking outdoors, taking a bath, spending time alone, staying positive	• Only female participants were sought and included. • Five of the participants interviewed with their parents in the room, which may have biased their responses. • Youth ethnicity/race not reported

### Data Synthesis

Data synthesis of the extracted findings was performed by two authors (R.L.W. and J.P.) using the thematic synthesis approach for qualitative research in systematic reviews.^69–72^ The first phase involved coding the text that was isolated during data extraction.^69^ To do so, each reviewer used line-by-line coding to capture the meanings of the studies’ findings. Following this, the next phase of data synthesis involved organizing the codes into descriptive themes.^69^ This allowed us to start grouping codes together by looking for similarities and differences. Additional codes were generated to label these groups. The final stage involved developing analytical themes.^69^ This stage intends to go beyond the findings of the primary studies that were included to address the research questions of this systematic review. This was initially done independently by each reviewer (R.L.W., A.B., and J.P.) and then discussed as a group. Through discussion, the final themes emerged when a consensus was reached among the team. These final themes are denoted as the “synthesized findings.”

## Findings

A total of 20 studies were included in this systematic review. From these, two synthesized findings with 24 subfindings were identified (see Figure 2). The studies that were included in this review largely focused on how young people experienced living with chronic pain and the ways in which they coped and managed their lives with their chronic pain. Though our aim was to report on experiences of youth living with chronic pain both with and without a formal diagnosis, the studies in this review did not highlight any differences or similarities between the experiences of these groups. All of the studies included mention of coping strategies within their qualitative findings, despite not being an initial aim for some studies.

Participants in the studies ranged from 6 to 24 years old. The studies were mostly conducted in Canada (*n* = 6), the United Kingdom (*n* = 5), and the United States (*n* = 3). Most the studies included participants with a variety of chronic pain conditions; others included participants with a specific condition and co-occurring chronic pain, such as cerebral palsy, complex regional pain syndrome, juvenile arthritis, and chronic headaches. Out of all the papers 14 included participants who had chronic pain associated with a co-occurring condition. Through these studies, the voices of 302 youth with chronic pain are represented.

Key findings of the systematic review indicated that young people with chronic pain experienced life much differently than their peers, which is identified through the first synthesized finding, *A Different Way of Being*. Chronic pain impacted youths’ relationships with their families and friends, and it altered their education and plans for the future. It also changed their sense of self-identity. To manage their chronic pain, youth had to find effective methods of coping, which are highlighted in the synthesized finding *Learning to Get By*. These coping strategies included both self-directed measures and external supports. Self-directed coping measures were activities that youth could do themselves to help manage their pain. External supports were methods of coping in which youth utilized outside help, such as that from a family member or a health care professional.

### Synthesized Finding 1: A Different Way of Being

*A Different Way of Being* signifies what it means for youth to live with chronic pain. Living with chronic pain impacted not only their way of life but also their identity. Youth with chronic pain experienced changes in their bodies and minds because of the symptoms associated with chronic pain, which, in turn, impacted their way of being in the world. Ten findings from two different categories made up the first synthesized finding of *A Different Way of Being*.

The first and largest category is *Disruption of Life*. This category indicates how common it was for youths’ lives to be disrupted and changed by chronic pain. At times, chronic pain was an all-consuming experience that disrupted countless aspects of their lives. As one youth described it:

But at the time when you’re in pain and everything you don’t think about anybody else! You think, “Oh my God, it’s hurting me, and this might be the worse it can get. Like this feels really bad for me!” ’Cos at the time … you don’t think that anyone else can be in worse pain as you’ve got.^50(p757)^

The experience of youth being in total pain, which included spiritual, emotional, social, and psychological pain alongside physical pain, was also reported.^62^ Experiencing pain in all aspects of their lives and having few long-term solutions available to them was a distressing experience for youth that had a significant impact on their mental health. Some youth reported hopelessness, depression, anxiety, and suicidal thoughts due to their chronic pain.^62^

Youth found that their lives were structured around pain, with one youth sharing that pain “controlled most of my decisions over the past month.”^57(p7)^ Youth also discussed the reality of having a lack of independence due to their chronic pain, with one youth sharing:

I try to do everything I want to do, but some things will stop me. When I’m a bit older I hope I can do, I’d like to be able to do whatever I like without thinking … like, is it going to hurt me or whatever.^50(p759)^

Chronic pain resulted in youth having to alter their participation in activities.^55^ Some youth reported that they frequently had to sit out of activities or they had to give up activities they once loved due to their chronic pain. One youth shared their experience with trying to stay active, stating, “I can’t pack it in because I get really, really tired and tired just doesn’t help the pain and then this whole spiral effect starts to happen.”^52(p169)^

Further, youth noted changes in their relationships with their friends, with some of their friends not being there for them.^50^ One youth shared, “Like, a proper friend will stand by you. … But some friends find it hard to cope with. But they should help you.”^50(p759)^ These difficult shifts in relationships added to how chronic pain changed and disrupted young people’s lives.

Other disruptions to their lives youth reported included having to rely on family for everyday tasks. For some youth, it became increasingly difficult for them to do these tasks independently with their chronic pain. One youth shared:

I get a lot of support from my family, it’s a big family so … my brothers and sisters look out for me. It’s been hard on them. They’ve had it quite hard. They’ve tried to make me comfy and get people to help you. … It’s quite difficult living with chronic pain.^50(p759)^

Another disruption youth shared was trouble keeping up with schoolwork. One youth highlighted, “My pain has stopped me from coming into school or being social in school. For example, I may be in a lot of pain and won’t want to be a bother to anyone else.”^57(p114)^

Because many youth with chronic pain missed school, could not participate in activities with peers, and were often exhausted from their pain, they tended to experience isolation. One youth described this difficult experience:

Since I was always in pain I did not want to go out with my friends or go to the football games or basketball games; I began to retreat within myself and in a sense hide out in my room. All I ever wanted to do was sleep; while I was sleeping I was not only avoiding life’s problems but it was the only time in which I wasn’t in pain.^64(p375)^

Youth with chronic pain reported frustration around the uncertainty of their future. Some youth also reported that this uncertainty with their chronic pain caused them to feel powerless, with one youth sharing, “The physical pain can be unbearable, but the psychological pain I get from living with all of these unknowns is so much crazier.”^68(p474)^ Changing future plans was also discussed as a disruption to their lives, with many youth fearing they would not be able to achieve their goals with their chronic pain onboard. Some youth reported that when they did have to change their plans, it felt like a loss due to their chronic pain. However, others felt positive about their future with chronic pain and developed new goals and plans that they could manage with their pain.^68^

The second category, *Identity and Pain*, refers to how youths’ identities had been impacted by their chronic pain. This category was made up of two findings. The first finding highlights how pain was unique to each person. Youth reported that their personal experience with chronic pain was specific and unique to themselves.^50^

Youths’ identities had been further impacted by seeing themselves as different from their peers. Youth with chronic pain reported being absent from school, needing to leave class, having to get up to stretch, and/or an inability to participate in physical education class, which made them feel like they were different from those around them.^51^ One youth shared their experience with being isolated from their peers, stating:

It has made me feel very disconnected, but I didn’t have the mental strength to do much about it. I know that once a bad period passes, they’ll [friends] be supportive, knowing I didn’t isolate myself on purpose, and I’ll be able to socialise normally. But it is quite depressing in the midst of isolation, being alone quite literally with my pain.^57(p114)^

Youth also felt that their pain has matured them, in comparison with their peers, with one youth sharing:

Adults think I’m more mature than most people and I think that might have something to do with having to be more responsible in think about, well, I can’t do that because that’ll make that happen. I can’t just go and do it, and deal with the consequence later because it’s too painful.^53(p118)^

Alongside this, youth reported a desire to feel “normal” among their peers. One youth shared their struggle with trying to hide their pain to avoid being treated differently at school, noting, “Well, I didn’t want to be treated like sort of, erm, I don’t know, make a big deal about it ’cause they would probably say do you want someone to write for you and I didn’t really want anyone to write for me.”^54(p103)^ These aspects of chronic pain impacted youths’ identities, adding to how living with chronic pain contributed to a different way of being.

### Synthesized Finding 2: Learning to Get by

The second synthesized finding is *Learning to Get By*. This includes how young people managed and coped with living with their chronic pain and discusses the interventions that youth did, as well as what other people did or did not do, to help youth get by. *Learning to Get By* is made up of 15 findings and two categories (see Table 2).

**Table 2.** Coping strategies identified in learning to get by.

Coping strategies	Type of pain	Cited in
Self-directed measures		
Medication	Not specified	Alsaggaf and Coyne^63^
Medication	Not specified	Forgeron and McGrath^57^
Medication	Juvenile idiopathic arthritis, type of pain not specified	Ghio et al.^62^
Medication	Cerebral palsy with abdominal pain, back pain, limb pain, face/temple/jaw pain, head pain, throat/neck pain, chest pain, groin/pubic pain	McKinnon et al.^66^
Medication	Not specified	Meldrum et al.^64^
Medication	HIV with chronic pain, type of pain not specified	Nkhoma et al.^54^
Medication	Not specified	Root and Nosek^58^
Medication	Not specified	Shaygan and Jaberi^65^
Medication	Chronic tension-type headache	Skogvold and Magnussen^67^
Heat pack	Not specified	Alsaggaf and Coyne^63^
Heat pack	Cerebral palsy with head, lower limbs, back, abdomen, face/jaw/temple pain	McKinnon et al.^66^
Heat pack	Not specified	Shaygan and Jaberi^65^
Massage	Not specified	Alsaggaf and Coyne^63^
Massage	Cerebral palsy with abdominal pain, back pain, limb pain, face/temple/jaw pain, head pain, throat/neck pain, chest pain, groin/pubic pain	McKinnon et al.^66^
Relaxing and resting	Not specified	Alsaggaf and Coyne^63^
Relaxing and resting	Not specified	Carter et al.^53^
Relaxing and resting	Back and lower limb pain	Castle et al.^60^
Relaxing and resting	Juvenile idiopathic arthritis, type of pain not specified	Ghio et al.^62^
Relaxing and resting	Not specified	Hurley-Wallace et al.^69^
Relaxing and resting	Cerebral palsy with back pain, limb pain, face/temple/jaw pain, head pain	McKinnon et al.^66^
Relaxing and resting	Not specified	Meldrum et al.^64^
Relaxing and resting	Not specified	Root and Nosek^58^
Relaxing and resting	Chronic tension-type headache	Skogvold and Magnussen^67^
Sticking to a routine	Chronic tension-type headache	Skogvold and Magnussen^67^
Distractions	Sickle cell anemia Celiac disease Crohn’s disease	Alsaggaf and Coyne^63^
Distractions	Pelvic pain Shoulder pain Neck pain Hand pain Foot pain Knee pain Stomach pain	Forgeron and McGrath^57^
Distractions	Type of pain not specified, all had co-occurring obesity	Gremillion et al.^6^
Distractions	Not specified	Jones et al.^55^
Distractions	Cerebral palsy, type of pain not specified	McKinnon et al.^66^
Distractions	HIV with chronic pain, type of pain not specified	Nkhoma et al.^54^
Distractions	Complex regional pain syndrome and other unspecified chronic pain conditions	Parsons et al.^68^
Distractions	Not specified	Shaygan and Jaberi^65^
Distractions	Not specified	Stinson et al.^72^
Distractions	Not specified	Szwimer et al.^59^
Exercise	Systemic lupus erythematous Chronic sinusitis Crohn’s disease	Alsaggaf and Coyne^63^
Exercise	Juvenile idiopathic arthritis, type of pain not specified	Ghio et al.^62^
Exercise	Not specified	Hurley-Wallace et al.^69^
Exercise	Not specified	Killackey et al.^70^
Exercise	HIV with chronic pain, type of pain not specified	Nkhoma et al.^54^
Exercise	Not specified	Root and Nosek^58^
Exercise	Chronic tension-type headache	Skogvold and Magnussen^67^
Exercise	Not specified	Szwimer et al.^59^
Pushing through the pain	Not specified	Carter et al.^53^
Pushing through the pain	Juvenile idiopathic arthritis, type of pain not specified	Ghio et al.^62^
Pushing through the pain	Not specified	Meldrum et al.^64^
Positive thinking	Sickle cell anemia Celiac disease Chronic sinusitis Systemic lupus erythematous Osteogenesis imperfecta	Alsaggaf and Coyne^63^
Positive thinking	Not specified	Shaygan and Jaberi^65^
Positive thinking	Not specified	Szwimer et al.^59^
Acceptance	Not specified	Szwimer et al.^59^
External supports		
Family	Not specified	Alsaggaf and Coyne^63^
Family	Not specified	Carter et al.^53^
Family	Cerebral palsy, type of pain not specified	Castle et al.^60^
Family	Juvenile idiopathic arthritis, type of pain not specified	Ghio et al.^62^
Family	Type of pain not specified, all had co-occurring obesity	Gremillion et al.^56^
Family	Not specified	Killackey et al.^70^
Family	Primary chronic pain, not specified	Mahon and Reynolds^71^
Family	Cerebral palsy, type of pain not specified	McKinnon et al.^66^
Family	HIV with chronic pain, type of pain not specified	Nkhoma et al.^54^
Family	Not specified	Shaygan and Jaberi^57^
Friendships	Not specified	Forgeron and McGrath^61^
Friendships	Not specified	Forgeron et al.^56^
Friendships	Type of pain not specified, all had co-occurring obesity	Gremillion et al.^56^
Friendships	Not specified	Jones et al.^55^
Friendships	Primary chronic pain, not specified	Mahon and Reynolds^71^
Friendships	HIV with chronic pain, type of pain not specified	Nkhoma et al.^62^
Friendships	Not specified	Shaygan and Jaberi^65^
Friendships	Not specified	Stinson et al.^72^
Friendships	Not specified	Root and Nosek^58^
Friendships	Not specified	Forgeron et al.^61^
Online communities and resources	Not specified	Hurley-Wallace et al.^69^
Seeking care	Not specified	Stinson et al.^72^
Seeking care	Cerebral palsy, type of pain not specified	Castle et al.^60^
Seeking care	Juvenile idiopathic arthritis, type of pain not specified	Ghio et al.^62^
Seeking care	Cerebral palsy, type of pain not specified	McKinnon et al.^66^
Seeking care	Not specified	Killackey et al.^70^
Seeking care	Not specified	Carter et al.^53^
Seeking care	HIV with chronic pain, type of pain not specified	Nkhoma et al.^54^
Seeking care	Not specified	Hurley-Wallace et al.^69^
Seeking care	Primary chronic pain, not specified	Mahon and Reynolds^71^
Seeking care	Not specified	Stinson et al.^72^

The first and largest category identified was *Self-Directed Measures*, which was made up of ten findings. Taking pain medication was one of the most notable self-directed coping methods for young people dealing with chronic pain. Although medication was not always helpful, youth often used medication as a first-line response to manage their chronic pain.^49,52,61,65^

A number of nonpharmacological methods of coping with chronic pain were also identified by youth. Using a heat pack applied to an area causing pain was effective for some youth.^49,60,65^ Massage was another self-directed coping method that was brought up by youth as useful way of managing their pain. Youth did not report massages as a suggestion from health care professionals. Youth shared that massages were an effective short-term method to managing pain, especially for youth with high levels of functioning.^49,60^

Relaxing was another frequently mentioned self-directed measure that helped youth cope with their chronic pain. Many youth turned to lying down, sleeping, and meditation to relax and manage their pain. Relaxing was reported to help provide comfort for youth while waiting for their pain to pass or lessen.^66^ As one youth with chronic arthritis pain stated, “I just tried not to move it, tried to keep it still. I did not do much because I was in pain. I couldn’t do many things because I was in pain.”^54(p102)^ Youth also reported having to rest or sleep often, especially on bad pain days, because their energy levels were often low.^57^ Adequate sleep was cited as an integral part of relieving pain and fatigue during the day, with youth citing early and consistent bedtimes being an important part of their pain management strategy.^66^

Other youth stated that to manage their pain they needed to follow a strict routine. Sticking to a routine helped youth prevent random pain episodes and allowed them to keep a consistent sleep routine, which was key in managing their pain.^66^

Youth also identified distractions, such as focusing on hobbies or hanging out with friends, as a useful self-directed method of coping with their pain.^63^ As one youth said, “Distraction is my main thing. Anything to get my mind off of it.”^52(p169)^ Engaging in these activities helped youth occupy their minds and keep their focus off of their pain. This was highlighted by one youth with chronic pain, who stated, “Reading is my happy place when I need to relax. I enter inside my book and forget about my pain for a while. I keep myself occupied so that the pain does not invade my thoughts.”^68(p472)^

Exercise was another self-directed measure that young people brought up. At times, intense exercise was difficult for youth with chronic pain. As such, some youth found modified or gentle exercise to be an effective way of dealing with pain.^66^ This was mentioned by one youth with chronic pain, who noted, “I had to adapt by choosing different sports than the ones I did before, but I enjoy these new sports a lot.”^68(p473)^

In contrast, youth commonly pushed through their pain as a self-directed method of coping. Although difficult for some, pushing through the pain helped young people try to live a relatively normal life and keep up with their activities, friends, and school. As noted by one youth in regard to participating in physical activities, “Get on with it, try not to think about it and if it’s really sore put something on it that helps like cool it down or stop it from feeling as much pain.”^54(p102)^

Positive thinking was another self-directed coping method mentioned by youth. Positive thinking helped youth believe that their pain was not permanent and that it would eventually end. As one youth said, “When we are in pain, we must think about good things, or the fact that we can carry on living.”^65(p7)^ Youth reported the value of maintaining hope that one day they will recover from their chronic pain and live like their healthy peers.^53^ Although many young people accepted that they might never live a pain-free life, many hoped to live with less pain one day. As one youth stated, “I must not lose hope. I should not be indifferent, and I should try to make the pain better.”^65(p7)^ Youth also created safe internal spaces for themselves as a way to cope with their pain. Being able to go to a place within the self to find peace and comfort helped to keep pain off their minds.

Finally, acceptance of their pain was found to be a useful self-directed coping measure for some youth. Learning to accept that their pain was most likely incurable helped youth maintain a more positive outlook. As one youth stated, “I can either mope about my pain, or I could suck it up, move on, and get on with life and stop letting it bring me down.”^68(p473)^ Arriving at a point of acceptance helped youth restructure their lives in ways that led them to develop a sense of self with their pain and allowed them to focus more on how to live a fulfilling life with their chronic pain.^68^ As one youth shared, “I realize that I cannot control my pain and it won’t just go away. I control how I live and deal with it.”^68(p473)^

*External Supports*, which describes the different supports youth accessed to manage their chronic pain, was the other category of *Learning to Get By*. This category is made up of four findings. Youth sought external support through family members, friends, and online communities, as well as through seeking care from professionals in the health care system.

Support from families was stated as an important external support for many youth. As one youth discussed, “I used to stay home on my own when my Mum went for a holiday or for a weekend away. … I didn’t need any care. Whereas now, constantly I need someone to be with me and that is really frustrating when you’re nearly 21.”^51(p448)^ Family support played a primary role in youths’ care, alongside providing them with meaningful social engagement. Because many young people with chronic pain are unable to participate in activities with their peers, time spent with their family could serve as one of their main forms of socialization.^60^ Support from their family also allowed youth an opportunity to talk about their pain with others, which helped them to cope. As one youth shared, “Normally I just try to talk to someone. Talking to my mum or dad.”^62(p1132)^

Friendships were also identified as an important external support for many youth. They relied on friends for emotional support and for support in school. As one youth stated, “My friends to create a happy environment for me, and help me walk, sit down and do my studies. For example, buy me things from the school cafeteria, help me go up the stairs, and bear with me when I’m in a lot of pain.”^65(p8)^ Having friends around who could provide support was valuable for youth with chronic pain, but it was also acknowledged that friends were only helpful to a certain point, because many did not fully understand youths’ pain, which could occasionally lead to frustration.^52^

At the same time, many youth reported that their friends did not understand them and their chronic pain or that they felt misunderstood by their friends. As one youth presented, “I tend to avoid talking about having chronic pain because the uncommon times I do bring it up, I’m usually met with a complete misunderstanding which is really frustrating.”^64(p5)^ Other youth tried to mask their pain from their peers. As one youth mentioned:

I don’t really explain about the pain, I’d rather not put it out there, and then—because people change things around and they’d probably make up weird stories about me. I don’t really like to talk to them about it, because I don’t know, I kind of have the fear that if I do, they’re going to treat me different. And I don’t want that.^53(p119)^

Having friends who also had chronic pain provided a unique means of external support for youth with chronic pain. Peers understood the experience of having chronic pain, and this helped youth feel less alone in their experiences. On the value of having friends with chronic pain, one youth shared:

Oh my gosh, she has made a world of difference in my life. I found talking with her helped. I could tell her anything. I didn’t think it would be that awesome or great to have someone with pain to talk to, [but] that’s what really helps me—not feeling so alone.^52(p170)^

Online communities and resources were also found to be a key external support for youth with chronic pain. Online communities provided youth with the opportunity to get advice and information from other people who live with their same condition. They also provided a space for youth to share articles, new treatments, and experiences with people from around the world who have similar conditions.^56^

Seeking care from the health care system was found to be another external support for youth with chronic pain. Youth sought out doctors as an external support for advice on managing their pain and to obtain necessary medications.^58,62^ Engaging in care through formal physiotherapy was cited by some youth to be a core pillar of their pain management.^66^ Talking to a psychologist about their pain was another external outlet within the health care system that young people found helpful for coping. This was noted by one youth, who stated, “I’ve been able to talk … me and me mum come to see (the psychologist), she works here, once a week like on the counselling side of it all.”^50(p759)^

However, despite this assistance with coping, youth with chronic pain described seeking professional help through the health care system as exhausting. Many youth experienced going between a myriad of different health care providers and clinics to access care and tests, which was often mentally and physically taxing.^51^

In their interactions with the health care system, youth noted the benefits of receiving a formal diagnosis for their pain while seeking care. It helped to legitimize the pain they were experiencing, reinforced that there was a reason for their pain, and helped them to receive proper treatment.^56^ Having a diagnosis also helped youth seek out specific pain management strategies, which aided in coping with their chronic pain.^56^ However, the journey to receiving a diagnosis was often challenging and lengthy for youth with chronic pain and, even then, youth did not always end up with a diagnosis. For some youth, these challenges and a lack of answers pushed them to do their own research on their symptoms to support obtaining a diagnosis:

I found obviously the NHS [National Health Service] page and just reading all the symptoms and it was just all adding up and “could it be something like this?” because it’s not like you can get a blood test for it and the doctors could just miss it. So, I booked an appointment, and I didn’t mention the fibromyalgia because I didn’t want to put something in the doctor’s head that might not be the case, but when I went to the doctor, I said about all my symptoms and the first thing he said was, “fibromyalgia.”^56(p347)^

In addition, young people shared that once they received a diagnosis, they often found that their access to appropriate external supports through the health care system remained difficult. It was not simple to access proper treatment to help them cope with their pain, and health care professionals continued to doubt them when they were in pain. As one youth with a diagnosis of primary chronic pain recalled:

I’ve been called crazy by doctors. I’ve had doctors be, it’s in your head, and you’re crazy. There’s something wrong with you. And so being a 10-year-old telling my mother that I have this problem and then going to the doctor then being, “your kids making this up this is crazy”. That’s hard for a kid hear and to deal with.^59(p21)^

Further, youth reported facing barriers facing barriers seeking care. This included difficulty obtaining information from their doctor about their diagnosis and challenges with finding relevant and reliable information about their diagnosis from other sources on the internet.^67^ Some reported receiving inappropriate treatment for their pain. The ability to access a pain clinic was also shown to be challenging for many youth, with some youth having to wait years until they could access a clinic and proper care.^59^ These barriers made it difficult for youth to fully understand their diagnosis and the treatments available for them and to develop coping habits, which could be a frustrating experience while they were in pain.

## Discussion

This review aimed to synthesize the experiences of youth living with chronic pain and the various coping methods they used to manage their chronic pain. To our knowledge, this is one of the only systematic reviews that examines the coping strategies of youth with chronic pain explored through qualitative studies. We reviewed 20 studies and arrived at two synthesized findings. It is apparent that youths’ lives have been significantly impacted by chronic pain. Pain was an all-consuming experience for many young people, impacting countless aspects of their everyday lives and affecting them emotionally and physically.^50,62^

Pain has a large impact on the whole person. As identified in this review, youths’ identities and sense of self were heavily shaped by their chronic pain.^50^ Chronic pain can present specific developmental challenges for youth.^73^ For instance, encouraging and supporting independence and autonomy is essential for the development of emotional intelligence and self-esteem in young people.^74^ Similarly, in this life stage, youth rely heavily upon peer support for their psychosocial development, because they typically transition to spending more time with friends and less with family.^75^ For youth with chronic pain, these aspects of development can be disrupted, as noted in this review. Adolescence is also a sensitive time for mental health and well-being, because mental health challenges often arise during this developmental period.^75^ This is of importance because youth with chronic pain are at an elevated risk for developing mental health conditions, such as depression and anxiety.^7^ As youth with chronic pain move into adulthood, some of the challenges in living with chronic pain may result in youth experiencing socioeconomic disparities, including lower educational attainment and a greater likelihood of relying on social support benefits.^33^ A person-centered approach to supporting youth with chronic pain is essential to strengthen their development and address the needs that are unique to young people.^73^

Youths’ coping methods are often unique to their age group, because they largely focus on their social and emotional well-being.^76^ Youth in this review developed their own self-directed measures of coping with their pain and, by doing so, often ended up taking on the majority of the coping burden themselves. These self-directed coping methods tended to be nonpharmacological, unique to the individual, and often arose from trial and error. Youth should feel empowered to manage their pain independently, and by using shared decision making with health care providers, youth can be encouraged to utilize the healthy coping strategies that work best for them.^77^ Similarly, health care providers can support youth by providing them with advice and patient education on coping strategies that could be helpful for youth to manage their chronic pain.^58^

Throughout the review, it was noted that there were limited coping frameworks available based on the grounded experience of youth living with chronic pain, with only one article included in this review that developed and used a coping framework based on the input of young people.^54^ By incorporating this framework into their study, the researchers were able determine specific trends among the experiences of youth with chronic pain, such as its impacts on their social lives and families.^54^ More work needs to be done to create frameworks that specifically include the experiences of youth with chronic pain.

Having supports was a key aspect in helping youth effectively cope with their chronic pain. Yet, from this review, it was apparent that there were few formal supports available for youth with chronic pain. This made life with chronic pain difficult for youth, who often had to rely heavily on supports available to them through their interpersonal connections and relationships to help them manage their pain.^64^ Of note, friends and family members were often the individuals who provided this support to youth to help them cope with their pain. However, not all youth with chronic pain have support networks available to them. Recognizing this, it is vital to integrate community-based supports to help promote functioning among young people with chronic pain. For example, formal peer support programs have shown promise in providing emotional support for youth with chronic pain.^78^

For youth who do have support networks, the quality of such networks can also vary. Because chronic pain is often not visible to others, this can lead to invalidation of the pain youth were experiencing and, consequently, stigmatization. Stigma occurs when others express disbelief regarding their pain, either directly or indirectly.^79^ Unlike acute pain, chronic pain can raise suspicions about the genuineness of the pain, particularly when it is not obvious to an onlooker or justifiable by a medical explanation.^80,81^ Young people have reported facing stigma related to their chronic pain from medical professionals, at school, and from peers and families.^57,78,81^ In attempts to reduce this stigma, youth may conceal the intensity and frequency of their pain from others, especially if others perceive the young person as being at fault for their chronic pain condition.^80,81^ To address stigma, public education is needed to inform and provide increased awareness about chronic pain and help shift beliefs and attitudes among the general public about the experiences of young people living with chronic pain.^2^

In addition to the external support they receive from their friends and family, youth with chronic pain require further external support from the health care system. Despite this, youth with chronic pain in our review experienced numerous challenges and barriers with the health care system, encountering care that was frequently not accessible, appropriate, or holistic. Throughout the review it became evident that accessing the health care system was often an ineffective coping method and had the potential to cause youth more distress.^59^ However, some health care supports provided effective means for coping, such as physiotherapy and counseling.^50,66^

Because the experience of pain is complex and unique to the individual, it is essential that care for youth with chronic pain actively involve the individual and considers their needs and preferences.^2^ Findings from this review show that care needs to take a whole-person and family-centered approach, with treatment and care considering physical and psychological strategies, in addition to pharmacological ones.^77^ By using a person-centered approach that includes the young person as an active member of their care, providers can facilitate the use of these strategies in ways that best meet youths’ coping needs.^82^ Beyond this, chronic pain can be complex and challenging to treat, requiring specialized knowledge of chronic pain management specific to young people.^83^ Even when youth have received a diagnosis and are receiving treatment, it can still be difficult for them to receive the appropriate health care and services they need.^62^

In this review, it was noted that the included studies did not highlight differences or similarities in chronic pain experiences between youth who had a formal diagnosis for their pain and those who did not. However, it was unclear whether this was a result of researchers not examining such differences or whether no differences were reported by the youth. Regardless of whether a youth has received a formal diagnosis, the pain they are experiencing is still real. This should be affirmed to youth and emphasized among providers.^59^ Youth with chronic pain need care and support that is built on trust and acceptance.^82^

In treating pain, health care providers often approach it by treating the level or intensity of pain reported by individuals. However, the recommended best practice involves addressing the functional impacts and emotional burden associated with chronic pain, as well as understanding the pain experience.^2,82^ The biopsychosocial model of pain acknowledges three main factors that contribute to one’s pain experience, and it is considered to be one of the most holistic models to examine and understand the basis of one’s chronic pain.^84^ Biological factors include any illnesses, injuries, or stressors that a person may be affected by and their genetic predisposition.^83^ Psychological factors include the emotional components tied to pain, as well as a person’s ability to feel active in managing their pain.^83^ Social factors consider how others involved in a person’s life respond to their pain, and this can also include experiences with their environments, such as their school life.^77,83^ Care for youth with chronic pain needs to be holistic, addressing the factors that influence their experience of pain, which include their biological, psychological, and social needs.^2,5^ As such, using the biopsychosocial model for understanding chronic pain care is critical, because it considers the whole person, their experience of pain, and how this pain impacts them.^85^ The biopsychosocial model also allows for recognition of the differences in pain experiences between young people and adults.^77^

One solution that has been proposed to enhance the care for youth with chronic pain is specialized pain clinics that are comprehensive and utilize an interdisciplinary approach.^86^ In such models of care, youth and their families are to be considered equal members of the care team and youth are actively involved in caring for their pain.^82^ Pain clinics that use the biopsychosocial model have expertise in treating chronic pain by addressing the impact of pain on the whole person, which enhances appropriate and holistic treatment for youth.^85^ Pain clinics are considered to be the gold standard for caring for people with chronic pain,^2^ and calls have been made to increase the accessibility of pain clinics that have a specialized focus on the pediatric population.^77,82^ Despite a clear need for comprehensive pain clinics for youth, such care is still lacking in Canada.^87^ Increased availability and capacity of pain clinics can help reduce issues with access and lengthy wait lists that youth in this review struggled with.^59^

Comprehensive chronic pain care should also address youths’ unique physical, emotional, and cognitive development states.^77^ In addition, the interplay of youths’ social relationships, environments, and mental health further impact their chronic pain experience.^77^ To address these factors, care should integrate multiple modalities.^77^ Combining pharmacological, physical, and psychological therapies together provides greater efficacy to treat chronic pain than singular therapies, and those that utilize cultural approaches suitable for the individual can further support youths’ outcomes.^2,77^

It was noted throughout this review that there was a need for more studies that connect health care access with its impacts on youth being able to cope with their chronic pain. Further research in this area could help affirm the need for specialized youth-specific chronic pain care and resources.^88^ More studies need to be done regarding the impacts of pain clinics on youths’ abilities to manage their pain and how this can further influence their daily lives with chronic pain. Having insight into these specific health care services could promote more youth-centered models of care and in turn improve the ways in which youth cope with their chronic pain.

## Limitations

There were numerous identified limitations to the papers that were reviewed. These papers did not break down the age ranges of participants in the presentation of their qualitative results, therefore limiting the knowledge of how coping methods were used across youth age groups. Another limitation was that there was no analysis of coping among different racial or ethnic groups, with few studies including such youth voices. There was also a lack of male and gender-diverse youth among participants in most of the papers included in the review. Further, it was found that only three papers included participants who did not have a formal diagnosis. These three studies also did not include separate categories of analysis for those with and without a formal diagnosis within their study, which is an area for future study. Another limitation that was identified was that none of the papers explored how pain clinics helped youth cope with their chronic pain. There is a need for additional qualitative studies grounded in youths’ experiences with a strict focus on youth coping with chronic pain, including further examination on the effectiveness of different coping methods, how youth coped with their pain based on their specific chronic pain conditions, and when they employed certain coping strategies. Such research will contribute to theory building specific to youth living with chronic pain. Lastly, only papers written in English were included, limiting the findings by excluding papers published in other languages.

## Conclusion

This systematic review aimed to synthesize insights from qualitative studies examining the experiences of youth with chronic pain and their coping strategies. From this, we arrived at two synthesized findings from 20 included studies. These synthesized findings highlighted the meanings youth assigned to living with their chronic pain and the complex experiences they have (*A Different Way of Being*), as well as the self-directed strategies they employed and the external supports they relied on from family, friends, online communities, and professionals within the health care system (*Learning to Get By*). This highlighted that young people have unique and complicated experiences living with chronic pain, diverse methods of coping with their chronic pain, and challenging interactions with the health care system and seeking proper care. Future research should be undertaken to examine the coping experiences of racial minority youth with chronic pain, as well as male and gender-diverse youth with chronic pain. Findings from this study can be used to support the well-being of youth living with chronic pain.

## Supplementary Material

ENTREQ Check List_December 17 2024.docx

Chronic Pain CP_Clean Copy_December 20.docx
