# Supplementary material for: Coping in youth living with chronic pain: A systematic review of qualitative evidence
Source: Can J Pain. 2025 Feb 25;9(1):2455494. doi: 10.1080/24740527.2025.2455494 (PMC11864317; doi:10.1080/24740527.2025.2455494)
Supplement: Chronic Pain CP_Clean Copy_December 20.docx [file UCJP_A_2455494_SM7861.docx]

**Coping in youth living with chronic pain: A systematic review of qualitative evidence**

Roberta L. Woodgate^a^* [ORCID ID: <https://orcid.org/0000-0002-7176-2390>]

Ashley Bell^a^ [ORCID ID: <https://orcid.org/0009-0001-1094-748X>]

Julianna Petrasko^a^ [ORCID ID: <https://orcid.org/0009-0005-4200-1604>]

Christine J Neilson^b^ [ORCID ID: <https://orcid.org/0000-0002-0525-737X>]

Olabisi Ayeni*^a^* [ORCID ID: <https://orcid.org/0009-0000-4888-0762nil>]

*^a^College of Nursing, Rady Faculty of Health Sciences, University of Manitoba, Winnipeg, Canada; ^b^Neil John Maclean Health Sciences Library, University of Manitoba, Winnipeg, Canada*

*Corresponding Author: Dr. Roberta L. Woodgate, College of Nursing, Rady Faculty of Health Sciences, University of Manitoba, 89 Curry Place, Winnipeg, MB R3T 2N2, Canada, Roberta.Woodgate@umanitoba.ca

**Coping in youth living with chronic pain: A systematic review of qualitative evidence**

**Abstract**

**Background:** Chronic pain is progressively receiving attention as a universal public health priority. It is anticipated that there will be an increase in the prevalence of chronic pain in the coming years, particularly among youth. Chronic pain can be stressful and have a significant impact on young people and their family.

**Aims:** The aim of this systematic review was to synthesize the best available qualitative evidence on the coping experiences of youth living with chronic pain and to note if there were any differences in their coping experiences.

**Methods:** A multi-database search was conducted including Child Development and Adolescent Studies, CINAHL, MEDLINE, PsycINFO, Embase and Scopus were searched for eligible English-language articles from inception to December 2023. Out of 1625 article titles and abstracts screened for eligibility, 280 articles underwent full-text screening, with 20 ultimately meeting all inclusion criteria. We conducted a thematic analysis of data extracted from the 20 reviewed articles.

**Results:** We arrived at two synthesized findings. *A Different Way of Being* considers the experience of being a youth with chronic pain. *Learning to Get By* looks at the coping strategies youth use to manage their chronic pain and involved youth using *self-directed strategies,* as well as relying on *external supports.*

**Conclusions:** It is apparent from these synthesized findings that youth’s lives have been significantly impacted by chronic pain. Findings from this study can be used to support the care and well-being of youth living with chronic pain.

**Keywords:** Chronic pain, youth, coping, systematic review, qualitative, youth experiences, health care system

**Word Count:** 7765 words

**Introduction**

Chronic pain is progressively garnering attention as a universal public health priority,^1,2^ having been included in the International Classification of Disease and Related Health Problems, a global classification systems of diseases and health related problems, in 2021.^3,4^ Defined as persistent or recurring pain that lasts more than 3 months, chronic pain is an unpleasant sensory experience that may or may not be associated with a physical injury.^3^ The experience of pain is unique to the individual and can be affected by biological, psychological, and social factors.^2,5^ Current research predicts an upsurge in the prevalence of chronic pain, especially among youth (i.e., young people aged 12-21 years).^6-9^

Chronic pain affects an estimated 20% of people worldwide,^3,10-12^ with a similar rate of 20.8% of youth globally experiencing chronic pain.^13^ These rates can vary, with a higher prevalence of chronic pain seen in youth who are girls,^13-17^ have a lower socioeconomic status,^14^ are a racial minority,^18^ and are older.^18^ Many youth report chronic headache, abdominal, back, and musculoskeletal pain,^5,13,14,16,17,19^and among youth who experience chronic pain, 20.6% note experiencing pain in at least two body sites.^16^

Chronic pain can be stressful, and its impact is pervasive. For youth with an undiagnosed chronic pain condition, this, alongside diagnostic uncertainty, can be stressful for youth and families.^20^ Even with a diagnosis, youth can experience confusion and uncertainty about their condition and how it will impact their life.^20^ Youth living with chronic pain can experience functional impairment and worse general well-being.^15,18^ They may also experience increased school absenteeism,^15,21^ anxiety,^7,14,22,23^ depression,^7,14,22-25^ and loneliness,^26^ as well as lower quality of life,^15,18^ and self-esteem.^14^ Chronic pain can impact the development of youth, and can contribute to mental health challenges and substance use disorders into their adulthood.^2,7,9,23,27,28^ Their pain experience often has a significant impact on their family members as well.^2,5^

Approximately 1 out of 3 youth with chronic pain struggle to cope with their pain.^25^ Coping, defined within the context of youth well-being, is the “conscious and volitional efforts to regulate emotion, cognition, behaviour, physiology, and the environment in response to stressful events or circumstances.”^29(p. 89)^ Youth can utilize a range and mix of different approaches to help them cope with their chronic pain, such as distraction, deep breathing exercises, and talking with others about their pain.^30,31^ Youth with chronic pain who are able to utilize effective coping strategies have improved quality of life.^31,32^ The effectiveness of such coping strategies also corresponds to youth’s pain levels, with coping strategies being more effective for lower levels of chronic pain.^31^

Little attention has been given to how youth cope with chronic pain, despite the long-term risks for social and economic disparities,^33^ and their persisting challenges into adulthood, such as higher levels of depression and anxiety.^7-9^  Reviews on coping with chronic pain among youth are often quantitative.^34^ While such reviews have contributed to the knowledge of coping with chronic pain, they lack the ability to explore the unique meanings behind chronic pain experiences**.** Existing qualitative syntheses of chronic pain in youth have explored the experiences of youth’s fluctuating pain in musculoskeletal disorders,^35^ examined youth’s experiences coping with chronic pain who had a history of psychological trauma,^36^ looked at the impact of chronic pain on youth’s school functioning,^21^ and the experiences of youth living with non-clinical chronic pain.^37^ At present, no qualitative systematic review captures the experiences of youth coping with chronic pain across diagnosed and undiagnosed conditions.

Considering this limitation, a synthesis of the literature focusing on the coping experiences of youth living with chronic pain will be useful to arrive at an increased understanding of their experiences, which can then be utilized to help us support youth’s health and well-being.^25^ Therefore, the aim of this systematic review was to synthesize the best available qualitative evidence on the lived experiences of youth living with chronic pain, with or without a formal medical diagnosis, with attention to how they cope. Specifically, the following questions were addressed:

1. What meanings do youth assign to living with chronic pain?
2. How do youth cope with their chronic pain?

**Methods**

This review is reported in accordance with the enhancing transparency in reporting the synthesis of qualitative research (ENTREQ) statement, which consists of 21 items for assessing the searching and selecting of qualitative research, quality appraisal, and methods for synthesizing qualitative findings (see supplemental material).^38^

A professional librarian (CJN) designed a draft search strategy to identify relevant literature in Ovid Medline with input from members of the research team. A second librarian peer reviewed the search using the 2015 PRESS guideline and checklist.^39^ The finalized search was translated and run in each of the following databases*:* Child Development and Adolescent Studies (EBSCO); CINAHL with Fulltext (EBSCO); Ovid MEDLINE(R) and Epub Ahead of Print, In-Process, In-Data-Review & Other Non-Indexed Citations and Daily; Embase (Ovid); APA PsycInfo (Ovid); and Scopus (Elsevier). A multi-database search was conducted in Sociological Abstracts and Social Services Abstracts via the Proquest platform. Searches were last updated in the databases listed above on December 4, 2023.

The search incorporated a modified version of DeJean and colleagues' qualitative research search filter to focus search results on qualitative and mixed methods research.^40^ An additional filter was used in Scopus to exclude Medline and Embase records from the Scopus search.^41^ No other limits or filters were used. Duplicate records were identified and removed from the initial result set using Endnote.^42^ Records identified when the search was updated were deduplicated by Covidence software.^43^ Complete search histories for each database are available in the University of Manitoba institutional repository.^44^

The criteria for studies to be included were: 1) it must be a qualitative study using any study method. Mixed method studies were included if the qualitative findings were included separately; 2) qualitative findings must include reports directly from youth themselves, and not through a proxy, such as a caregiver or parent; 3) strategies or management approaches for coping with chronic pain must be reported in the findings; 4) youth reporting must be between 12-21 years old (mean age of >12 or <21 if a broader age range sampled) and must live with chronic pain (defined as consistent or recurrent pain lasting longer than 3 months with or without a clear physical pathology or diagnosis); and 5) it must be published in a peer-reviewed English language journal. It should be noted that while youth is best understood as a period of transition from the dependence of childhood to adulthood’s independence, the age range does vary depending on the source.^45^ For the purposes of this review, the age range of 12 to 21 years was selected in recognition that chronic pain becomes prominent for many youth during adolescence^46^ and that around 21 years is when youth transition to adult based care.^47^

Studies were excluded if: 1) the complete study was not available; 2) the paper was a meta-analysis or meta-synthesis; 3) it was a different topic area; 4) it was not original research; 5) no qualitative findings were reported; 6) the findings were not reported by youth; 7) youth did not belong to the correct age group; 8) the paper reported on acute pain only; or 9) there were no findings on coping strategies.

The initial search of the databases identified 4965 articles (see Figure 1), which were then imported into Covidence. After duplicates were removed, 1625 studies remained. The article abstracts were then assessed by two reviewers (JP and OA), and articles were removed if they did not fit the inclusion criteria. This left 280 articles for further screening. The second screening consisted of three reviewers (RLW, JP, and OA) reading the full article and including articles based on the inclusion criteria. A total of 30 studies were included. After further discussion among the authors (RLW, AB, JP, and OA), ten articles were dropped from the review for not meeting the inclusion criteria, leaving 20 articles at this point.

[Insert ‘Figure 1. PRISMA Flow Diagram’ here]

***Quality assessment***

The Mixed Methods Appraisal Tool (MMAT), which provides guidance for appraising the methodological quality of qualitative, quantitative randomized controlled, quantitative non-randomized, quantitative descriptive, and mixed method studies, was utilized for the quality assessment.^48^ The methodological quality assessment was done by two reviewers (RLW and JP) independently for each paper included at this stage. A quality score was given to each study using the MMAT. For each section, either a value of 1 for “Yes” responses or 0 for “No” responses were given. Any disagreements from the quality assessment were reviewed and discussed by four reviewers (RLW, AB, JP, and OA) until a consensus was agreed upon. At this point any article that was found to not meet a high methodological standard was excluded from the study. All 20 articles that met the inclusion criteria also met the quality assessment standard, and, as such, all were included in this systematic review for data extraction.

***Data extraction***

Three reviewers (RLW, JP and OA) extracted the data relevant to the research aim and questions from the 20 studies (see Table 1.). The extracted data table included information pertaining to authors’ names, year published, country the study was conducted in, study design, purpose of the study, recruitment setting, age of the participating youth, data collection method, number of participating youth, participants’ type of chronic pain, main findings of the study, and limitations identified. Any disagreements during the extraction were discussed among three reviewers (RLW, AB, and JP) until an agreement was reached.

[Insert ‘Table 1. Characteristics of Studies in the Systematic Review’ here]

***Data* *synthesis***

Data synthesis of the extracted findings was performed by two authors (RLW and JP) using the thematic synthesis approach for qualitative research in systematic reviews.^69-72^ The first phase involved coding the text that was isolated during data extraction.^69^ To do so, each reviewer used line-by-line coding to capture the meanings of the studies’ findings. Following this, the next phase of data synthesis involved organizing the codes into descriptive themes.^69^ This allowed us to start grouping codes together by looking for similarities and differences. Additional codes were generated to label these groups. The final stage involved developing analytical themes.^69^ This stage intends to go beyond the findings of the primary studies that were included in order to address the research questions of this systematic review. This was initially done independently by each reviewer (RLW, AB, and JP), and then discussed as a group. Through discussion, the final themes emerged when a consensus was reached among the team. These final themes are denoted as the “synthesized findings.”

**Findings**

A total of 20 studies were included in this systematic review. From these, two synthesized findings with 24 sub-findings were identified (see Figure 2). The studies that were included in this review largely focused on how young people experienced living with chronic pain, and the ways in which they coped and managed their lives with their chronic pain. Though our aim was to report on experiences of youth living with chronic pain both with and without a formal diagnosis, the studies in this review did not highlight any differences or similarities between the experiences of these groups. All the studies included mention of coping strategies within their qualitative findings, despite not being an initial aim for some studies.

Participants in the studies ranged from 6 to 24 years old. The studies were most commonly conducted in Canada (n=6), the United Kingdom (n=5), and the United States of America (n=3). The majority of the studies included participants with a variety of chronic pain conditions, while others included participants with a specific condition and co-occurring chronic pain, such as cerebral palsy, complex regional pain syndrome, juvenile arthritis, and chronic headaches. Out of all the papers 14 had participants who had chronic pain associated with a co-occurring condition. Through these studies, the voices of 302 youth with chronic pain are represented.

[Insert ‘Figure 2. Summary of Synthesized Findings’ here]

Key findings of the systematic review indicated that young people with chronic pain experienced life much differently than their peers, which is identified through the first synthesized finding, *A Different Way of Being*. Chronic pain impacted youth’s relationships with their families and friend, and it altered their education and plans for the future. It also changed their sense of self-identity. To manage their chronic pain, youth had to find effective methods of coping, which are highlighted in the synthesized finding, *Learning to Get By*. These coping strategies included both self-directed measures and external supports. Self-directed coping measures were activities that youth could do themselves to help manage their pain. External supports were methods of coping in which youth utilized outside help, such as that from a family member or a health care professional.

***Synthesized finding 1: A different way of being***

*A Different Way of Being* signifies what it means for youth to live with chronic pain. Living with chronic impacted not only their way of life, but also their identity. Youth with chronic pain experienced changes in their bodies and minds because of the symptoms associated with chronic pain, which, in turn, impacted their way of being in the world. 10 findings from two different categories made up the first synthesized finding of *A Different Way of Being*.

The first and largest category is *Disruption of Life.* This category indicates how common it was for youth to have their lives disrupted and changed by chronic pain. At times, chronic pain was an all-consuming experience that disrupted countless aspects of their lives. As one youth described it:

But at the time when you’re in pain and everything you don’t think about anybody else! You think, ‘Oh my God, it’s hurting me, and this might be the worse it can get. Like this feels really bad for me!’ ’Cos at the time ... you don’t think that anyone else can be in worse pain as you’ve got.^50(p757)^

The experience of youth being in total pain, which included spiritual, emotional, social, and psychological pain alongside physical, was also reported.^62^ Experiencing pain in all aspects of their lives and having few long-term solutions available to them was a distressing experience for youth that had a significant impact on their mental health. Some youth reported hopelessness, depression, anxiety, and suicidal thoughts due to their chronic pain.^62^

Youth found that their life was structured around pain, with one youth sharing that pain “controlled most of my decisions over the past month.”^57(p7)^ Youth also discussed the reality of having a lack of independence due to their chronic pain, with one youth sharing:

I try to do everything I want to do, but some things will stop me. When I’m a bit older I hope I can do, I’d like to be able to do whatever I like without thinking ... like is it going to hurt me or whatever...^50(p759)^

Chronic pain resulted in youth having to alter their participation in activities.^55^ Some youth reported that they frequently had to sit out of activities, or they had to give up activities they once loved due to their chronic pain. One youth shared their experience with trying to stay active, stating, “I can’t pack it in because I get really, really tired and tired just doesn’t help the pain and then this whole spiral effect starts to happen.”^52(p169)^

Further, youth also noted changes in their relationships with their friends, with some of their friends not being there for them.^50^ One youth shared, “Like, a proper friend will stand by you… But some friends find it hard to cope with. But they should help you.”^50(p759)^ These difficult shifts in relationships added to how chronic pain changed and disrupted young people’s lives.

Other disruptions to their lives youth reported included having to rely on family for everyday tasks. For some youth, it became increasingly difficult for them to do these tasks independently with their chronic pain. One youth shared:

I get a lot of support from my family, it’s a big family so ... my brothers and sisters look out for me. It’s been hard on them. They’ve had it quite hard. They’ve tried to make me comfy and get people to help you ... It’s quite difficult living with chronic pain^50(p759)^

Another disruption youth shared was trouble keeping up with schoolwork. One youth highlighted, “My pain has stopped me from coming into school or being social in school. For example, I may be in a lot of pain and won't want to be a bother to anyone else.”^57(p114)^

As many youth with chronic pain missed school, could not participate in activities with peers, and were often exhausted from their pain, they tended to experience isolation. One youth described this difficult experience:

Since I was always in pain I did not want to go out with my friends or go to the football games or basketball games; I began to retreat within myself and in a sense hide out in my room. All I ever wanted to do was sleep; while I was sleeping I was not only avoiding life’s problems but it was the only time in which I wasn't in pain.^64(p375)^

Youth with chronic pain reported frustration around the uncertainty of their future. Some youth also reported that this uncertainty with their chronic pain caused them to feel powerless, with one youth sharing, “the physical pain can be unbearable, but the psychological pain I get from living with all of these unknowns is so much crazier.”^68(p474)^ Changing future plans was also discussed as a disruption to their lives, with many youth fearing they would not be able to achieve their goals with their chronic pain onboard. Some youth reported that when they did have to change their plans, it felt like a loss from their chronic pain. However, others felt positive about their future with chronic pain and developed new goals and plans that they could manage with their pain.^68^

The second category, *Identity and Pain*, refers to how youth’s identities had been impacted by their chronic pain. This category was made up of two findings. The first finding highlights how pain was unique to each person. Youth reported that their personal experience with chronic pain was specific and unique to themselves.^50^

Youth’s identities had been further impacted by seeing themselves as different from their peers. Youth with chronic pain reported being absent from school, needing to leave class, getting up to stretch, and/or an inability to participate in physical education class, which made them feel like they were different from those around them.^51^ One youth shared their experience with being isolated from their peers, stating:

It has made me feel very disconnected, but I didn't have the mental strength to do much about it. I know that once a bad period passes, they'll [friends] be supportive, knowing I didn't isolate myself on purpose, and I'll be able to socialise normally. But it is quite depressing in the midst of isolation, being alone quite literally with my pain.^57(p114)^

Youth also felt that their pain has matured them, in comparison with their peers, with one youth sharing:

Adults think I’m more mature than most people and I think that might have something to do with having to be more responsible in think about, well, I can’t do that because that’ll make that happen. I can’t just go and do it, and deal with the consequence later because it’s too painful.^53(p118)^

Alongside this, youth reported a desire to feel “normal” among their peers. One youth shared their struggle with trying to hide their pain to avoid being treated differently at school, noting, “well, I didn't want to be treated like sort of erm I don't know make a big deal about it 'cause they would probably say do you want someone to write for you and I didn't really want anyone to write for me.”^54(p103)^ These aspects of chronic pain impacted youth’s identities, adding to how living with chronic pain contributed to a different way of being.

***Synthesized finding 2: Learning to get by***

The second synthesized finding is *Learning to Get By.* This includes how young people managed and coped with living with their chronic pain and discusses the interventions that youth did, as well as what other people did or did not do, to help youth get by. *Learning to Get By* is made up of 15 findings and two categories (see Table 2).

[Insert ‘Table 2. Coping strategies identified in *Learning to Get By*’ here]

The first and largest category identified was *Self-Directed Measures*, which was made up of 10 findings. Taking pain medication was one of the most notable self-directed coping methods for young people dealing with chronic pain. Although medication was not always helpful, youth often used medication as a first-line response to manage their chronic pain.^49,52,61,65^

A number of non-pharmacological methods of coping with chronic pain were also identified by youth. Using a heat pack applied to an area causing pain was effective for some youth.^49,60,65^ Massage was another self-directed coping method that was brought up by youth as useful way of managing their pain. Youth did not report massages to be a suggestion from healthcare professionals. Youth shared that massages were an effective short-term method to managing pain, especially for youth with high levels of functioning.^49,60^

Relaxing was another frequently mentioned self-directed measure that helped youth cope with their chronic pain. Many youth turned to laying down, sleeping, and meditation to relax and manage their pain. Relaxing was reported to help provide comfort for youth while waiting for their pain to pass or lessen.^66^ As one youth with chronic arthritis pain stated, “I just tried not to move it, tried to keep it still. I did not do much because I was in pain. I couldn't do many things because I was in pain.”^54(p102)^ Youth also reported having to rest or sleep often, especially on bad pain days, as their energy levels were often low.^57^ Adequate sleep was cited as an integral part of relieving pain and fatigue during the day, with youth citing early and consistent bedtimes being an important part of their pain management strategy.^66^

Other youth stated that to manage their pain they needed to follow a strict routine. Sticking to a routine helped youth prevent random pain episodes and allowed them to keep a consistent sleep routine, which was key in managing their pain.^66^

Youth also identified distractions, such as focusing on hobbies or hanging out with friends, as a useful self- directed method of coping with their pain.^63^ As one youth said, “distraction is my main thing. Anything to get my mind off of it.”^52(p169)^ Engaging in these activities helped youth occupy their mind and keep their focus off of their pain. This was highlighted by one youth with chronic pain, who stated, “reading is my happy place when I need to relax. I enter inside my book and forget about my pain for a while. I keep myself occupied so that the pain does not invade my thoughts.”^68(p472)^

Exercise was another self-directed measure that young people brought up. At times, intense exercise was difficult for youth with chronic pain. As such, some youth found modified or gentle exercise to be an effective way of dealing with pain.^66^ This was mentioned by one youth with chronic pain, who noted, “I had to adapt by choosing different sports than the ones I did before, but I enjoy these new sports a lot.”^68(p473)^

In contrast, youth commonly pushed through their pain as a self-directed method of coping. Although difficult for some, pushing through the pain helped young people try to live a relatively normal life and keep up with their activities, friends, and school. As noted by one youth in regard to participating in physical activities, “Get on with it, try not to think about it and if it's really sore put something on it that helps like cool it down or stop it from feeling as much pain.”^54(p102))^

Positive thinking was another self-directed coping method mentioned by youth. Positive thinking helped youth believe that their pain was not permanent, and that it would eventually end. As one youth said, “when we are in pain, we must think about good things, or the fact that we can carry on living.”^65(p7)^ Youth reported the value of maintaining hope that one day they will recover from their chronic pain, and live like their healthy peers.^53^ Although many young people accepted that they might never live a pain-free life, many hoped to live with less pain one day. As one youth stated, “I must not lose hope. I should not be indifferent, and I should try to make the pain better.”^65(p7)^ Youth also created safe internal spaces for themselves as a way to cope with their pain. Being able to go to a within the self to find peace and comfort helped to keep pain off of their mind.

Finally, acceptance of their pain, was found to be a useful self-directed coping measure for some youth. Learning to accept that their pain was most likely incurable helped youth maintain a more positive outlook. As one youth stated, “I can either mope about my pain, or I could suck it up, move on, and get on with life and stop letting it bring me down.”^68(p473)^ Arriving at a point of acceptance helped youth restructure their lives in ways that led them to develop a sense of self with their pain and allowed them to focus more on how to live a fulfilling life with their chronic pain.^68^ As one youth shared, “I realize that I cannot control my pain and it won’t just go away. I control how I live and deal with it.”^68(p473)^

*External Supports,* which describes the different supports youth accessed to manage their chronic pain, was the other category of *Learning to Get By*. This category is made up of four findings. Youth sought external support through family members, friends, and online communities, as well as through seeking care from professionals in the healthcare system.

Support from families was stated as an important external support for many youth. As one youth discussed, “I used to stay home on my own when my Mum went for a holiday or for a weekend away… I didn’t need any care. Whereas now, constantly I need someone to be with me and that is really frustrating when you’re nearly 21.”^51(p448)^  Family support played a primary role in youth’s care, alongside providing them with meaningful social engagement. Since many young people with chronic pain were unable to participate in activities with their peers, time spent with their family could serve as one of their main forms of socialization.^60^ Support from their family also allowed youth an opportunity to talk about their pain with others, which helped them to cope. As one youth shared, “normally I just try to talk to someone. Talking to my mum or dad.”^62(p1132)^

Friendships were also identified as an important external support for many youth. They relied on friends for emotional support and for support in school. As one youth stated, “my friends to create a happy environment for me, and help me walk, sit down and do my studies. For example, buy me things from the school cafeteria, help me go up the stairs, and bear with me when I’m in a lot of pain.”^65(p8)^ Having friends around who could provide support were valuable for youth with chronic pain, but it was also acknowledged that friends were only helpful to a certain point, as many did not fully understand youth’s pain, which could occasionally lead to frustration.^52^

At the same time, many youth reported that their friends did not understand them and their chronic pain, or that they felt misunderstood by their friends. As one youth presented, “I tend to avoid talking about having chronic pain because the uncommon times I do bring it up, I’m usually met with a complete misunderstanding which is really frustrating.”^64(p5)^ Other youth tried to mask their pain from their peers. As one youth mentioned:

I don’t really explain about the pain, I’d rather not put it out there, and then – because people change things around and they’d probably make up weird stories about me. I don’t really like to talk to them about it, because I don’t know, I kind of have the fear that if I do, they’re going to treat me different. And I don’t want that.^53(p119)^

Having friends who also had chronic pain provided a unique means of external support for youth with chronic pain. Peers understood the experience of having chronic pain and this helped youth feel less alone in their experiences. On the value of having friends with chronic pain, one youth shared:

Oh my gosh, she has made a world of difference in my life. I found talking with her helped. I could tell her anything. I didn’t think it would be that awesome or great to have someone with pain to talk to, [but] that’s what really helps me—not feeling so alone.^52(p170)^

Online communities and resources were also found to be a key external support for youth with chronic pain. Online communities provided youth with the opportunity to get advice and information from other people who live with their same condition. They also provided a space for youth to share articles, new treatments, and experiences with people from around the world who have similar conditions.^56^

Seeking care from the health care system was found to be another external support for youth with chronic pain. Youth sought out doctors as an external support for advice on managing their pain and to obtain necessary medications.^58,62^ Engaging in care through formal physiotherapy was cited by some youth to be a core pillar of their pain management.^66^ Talking to a psychologist about their pain was another external outlet within the health care system that young people found helpful for coping. This was noted by one youth, who stated, “I’ve been able to talk ... me and me mum come to see (the psychologist), she works here, once a week like on the counselling side of it all.”^50(p759)^

However, despite this assistance with coping, youth with chronic pain described seeking professional help through the health care system as exhausting. Many youth experienced going between a myriad of different health care providers and clinics to access care and tests, which was often mentally and physically taxing.^51^

In their interactions with the health care system, youth noted the benefits of receiving a formal diagnosis for their pain while seeking care. It helped to legitimize the pain they were experiencing, reinforced that there was a reason for their pain, and helped them to receive proper treatment.^56^ Having a diagnosis also helped youth seek out specific pain management strategies, which aided in coping with their chronic pain.^56^ However, the journey to receiving a diagnosis was often challenging and lengthy for youth with chronic pain and, even then, youth did not always end up with a diagnosis. For some youth, these challenges and a lack of answers pushed them to do their own research on their symptoms to support obtaining a diagnosis:

I found obviously the NHS [National Health Service] page and just reading all the symptoms and it was just all adding up and “could it be something like this?” because it’s not like you can get a blood test for it and the doctors could just miss it. So, I booked an appointment, and I didn’t mention the fibromyalgia because I didn’t want to put something in the doctor’s head that might not be the case, but when I went to the doctor, I said about all my symptoms and the first thing he said was, “fibromyalgia”.^56(p347)^

Alongside this, young people shared that once they received a diagnosis, they often found that their access to appropriate external supports through the health care system remained difficult. It was not simple to access proper treatment to help them cope with their pain, and health care professionals continued to doubt them when they were in pain. As one youth with a diagnosis of primary chronic pain recalls:

I’ve been called crazy by doctors. I’ve had doctors be, it’s in your head, and you’re crazy. There’s something wrong with you. And so being a 10-year-old telling my mother that I have this problem and then going to the doctor then being, ‘your kids making this up this is crazy’. That’s hard for a kid hear and to deal with.^59(p21)^

Further, youth reported facing barriers facing barriers seeking care. This included difficulty obtaining information from their doctor about their diagnosis, and challenges with finding relevant and reliable information about their diagnosis from other sources on the Internet.^67^ Some reported receiving inappropriate treatment for their pain. The ability to access a pain clinic was also shown to be challenging for many youth, with some youth having to wait years until they could access a clinic and proper care.^59^ These barriers made it difficult for youth to fully understand their diagnosis, the treatments available for them, and to develop coping habits which could be a frustrating experience while they were in pain.

**Discussion**

This review aimed to synthesize the experiences of youth living with chronic pain and the various coping methods they used to manage their chronic pain. To our knowledge, this is one of the only systematic reviews that examines the coping strategies of youth with chronic pain explored through qualitative studies. We reviewed 20 studies and arrived at two synthesized findings. It is apparent that youth’s lives have been significantly impacted by chronic pain. Pain was an all-consuming experience for many young people, impacting countless aspects of their everyday lives and affecting them emotionally and physically.^50,62^

Pain has a large impact on the whole person. As identified in this review, youth’s identities and sense of self were heavily shaped by their chronic pain.^50^ Chronic pain can present specific developmental challenges for youth.^73^ For instance, encouraging and supporting independence and autonomy is essential for the development of emotional intelligence and self-esteem in young people.^74^ Similarly, in this life stage, youth rely heavily upon peer support for their psychosocial development, as they typically transition to spending more time with friends and less with family.^75^ For youth with chronic pain, these aspects of development can be disrupted, as noted in this review. Adolescence is also a sensitive time for mental health and well-being, as mental health challenges often arise during this developmental period.^75^ This is of importance as youth with chronic pain are at an elevated risk for developing mental health conditions, such as depression and anxiety.^7^ As youth with chronic pain move into adulthood, some of the challenges present living with chronic pain may result in them experiencing socioeconomic disparities, including lower educational attainment and a greater likelihood of relying upon social support benefits.^33^ A person-centered approach to supporting youth with chronic pain is essential to strengthen their development and address the needs that are unique to young people.^73^

Youth’s coping methods are often unique to their age group, as they largely focus on their social and emotional well-being.^76^ Youth in this review developed their own self-directed measures of coping with their pain, and by doing so, often ended up taking on the majority of the coping burden themselves. These self-directed coping methods tended to be non-pharmacological, unique to the individual, and often arose from trial-and-error. Youth should feel empowered to manage their pain independently, and by using shared decision making with health care providers, youth can be encouraged to utilize the healthy coping strategies that work best for them.^77^ Similarly, health care providers can also support youth by providing them with advice and patient education on coping strategies that could be helpful for youth to manage their chronic pain.^58^

Throughout the review, it was noted that there were limited coping frameworks available based on the grounded experience of youth living with chronic pain, with only one article included in this review that developed and used a coping framework based on the input of young people.^54^By incorporating this framework into their study, the researchers were able determine specific trends among the experiences of youth with chronic pain, such as its impacts their social life and family.^54^ More work needs to be done to create frameworks that specifically include the experiences of youth with chronic pain..

Having supports was a key aspect in helping youth effectively cope with their chronic pain. Yet, from this review, it was apparent that there were few formal supports available for youth with chronic pain. This made life with chronic pain difficult for youth and they often had to rely heavily on supports available to them through their interpersonal connections and relationships to help them manage their pain.^64^ Of note, friends and family members were often the individuals who provided this support to youth to help them cope with their pain. However, not all youth with chronic pain have support networks available to them. Recognizing this, it is vital to integrate community-based supports to help promote functioning among young people with chronic pain. For example, formal peer support programs have shown promise in providing emotional support for youth with chronic pain.^78^

For youth who do have support networks, the quality of such networks can also vary. As chronic pain is often not visible to others, this can lead to invalidation of the pain youth were experiencing, and consequently, stigmatization. Stigma occurs when others express disbelief regarding their pain, either directly or indirectly.^79^ Unlike acute pain, chronic pain can raise suspicions about the genuineness of the pain, particularly when it is not obvious to an onlooker or justifiable by a medical explanation.^80,81^ Young people have reported facing stigma related to their chronic pain from medical professionals, at school, and from peers and families.^57,78,81^ In attempts to reduce this stigma, youth may conceal the intensity and frequency of their pain from others, especially if others perceive the young person for being at fault for their chronic pain condition.^80,81^ To address stigma, public education is needed to inform and provide increased awareness about chronic pain and help shift beliefs and attitudes among the general public about the experiences of young people living with chronic pain.^2^

In addition to the external support they receive from their friends and family, youth with chronic pain require further external support from the health care system. Despite this, youth with chronic pain in our review experienced numerous challenges and barriers with the health care system, encountering care that was frequently not accessible, appropriate, or holistic. Throughout the review it became evident that accessing the health care system was often an ineffective coping method, and had the potential to cause youth more distress.^59^ However, some health care supports provided effective means for coping, such as physiotherapy and counselling.^50,66^

As the experience of pain is complex and unique to the individual, it is essential that care for youth with chronic pain actively involves the individual and considers their needs and preferences.^2^ Findings from this review show care needs to take a whole-person and family-centered approach, with treatment and care considering physical and psychological strategies, in addition to pharmacological ones.^77^ By using a person-centered approach that includes the young person as an active member of their care, providers can facilitate the use of these strategies in ways that best meet youth’s coping needs.^82^ Beyond this, chronic pain can be complex and challenging to treat, requiring specialized knowledge of chronic pain management specific to young people.^83^ Even when youth have received a diagnosis and are receiving treatment, it can still be difficult for them to receive the appropriate health care and services they need.^62^

In this review, it was noted that the included studies did not highlight differences or similarities in chronic pain experiences between youth who had a formal diagnosis for their pain and those who did not. However, it was unclear is this was a result of researchers not examining such differences, or if no differences were reported by the youth. Regardless of whether a youth has received a formal diagnosis, the pain they are experiencing is still real. This should be affirmed to youth and emphasized among providers.^59^ Youth with chronic pain need care and support that is built on trust and acceptance.^82^

In treating pain, health care providers often approach it by treating the level or intensity of pain reported by individuals. However, the recommended best practice involves addressing the functional impacts and emotional burden associated with chronic pain, as well as understanding the pain experience.^2,82^ The biopsychosocial model of pain acknowledges three main factors that contribute to one’s pain experience, and it is considered to be one of the most holistic models to examine and understand the basis of one’s chronic pain.^84^ Biological factors include any illnesses, injuries, or stressors that a person may be affected by, and their genetic predisposition.^83^ Psychological factors include the emotional components tied to pain, as well as a person’s ability to feel active in managing their pain.^83^ Social factors consider how others involved in a person’s life respond to their pain, and this can also include experiences with their environments, such as their school life.^77,83^ Care for youth with chronic pain needs to be holistic, addressing the factors that influence their experience of pain, which include their biological, psychological, and social needs.^2,5^ As such, using the biopsychosocial model for understanding chronic pain care is critical, as it considers the whole person, their experience of pain, and how this pain impacts them.^85^ The biopsychosocial model also allows for recognition of the differences in pain experiences between young people and adults.^77^

One solution that has been proposed to enhance the care for youth with chronic pain is specialized pain clinics for youth that are comprehensive and utilize an interdisciplinary approach.^86^ In such models of care, youth and their families are to be considered equal members of the care team, and youth are actively involved in caring for their pain.^82^ By utilizing the biopsychosocial model, pain clinics have expertise in treating chronic pain by addressing the impact of pain on the whole person, which enhances appropriate and holistic treatment for youth.^85^ Pain clinics are considered to be the gold standard for caring for people with chronic pain,^2^ and calls have been made to increase the accessibility of pain clinics that have a specialized focus on the pediatric population.^77,82^ Despite a clear need for comprehensive pain clinics for youth, such care is still lacking in Canada.^87^ Increased availability and capacity of pain clinics can help reduce issues with access and lengthy waitlists that youth in this review struggled with.^59^

Comprehensive chronic pain care should also address youth’s unique physical, emotional, and cognitive development states.^77^ As well, the interplay of youth’s social relationships, environments, and mental health further impact their chronic pain experience.^77^ To address these factors, care should integrate multiple modalities.^77^ Combining pharmacological, physical, and psychological therapies together provide greater efficacy to treating chronic pain than singular therapies, and those that utilize cultural approaches suitable for the individual can further support youth’s outcomes.^2,77^

It was noted throughout this review that there was a need for more studies that connect health care access with its impacts on youth being able to cope with their chronic pain. Further research in this area could help affirm the need for specialized youth-specific chronic pain care and resources.^88^ There also needs to be more studies done regarding the impacts of pain clinics on youth’s ability to manage their pain and how this can further influence their daily lives with chronic pain. Having insight into these specific health care services could promote more youth-centered models of care, and in turn improve the ways that youth cope with their chronic pain.

**Limitations**

There were numerous identified limitations to the papers that were reviewed. These papers did not break down the age brackets of participants in the presentation of their qualitative results, therefore limiting the knowledge of how coping methods were used across youth age groups. Another limitation was that there was no analysis of coping among different racial or ethnic groups among the papers, with few studies including such youth voices. There was also a lack of male and gender diverse youth among participants in the majority of the papers included in the review. Further, it was found that only three papers throughout the review included participants who did not have a formal diagnosis. These three studies also did not include separate categories of analysis for those with and without a formal diagnosis within their study, which is an area for future study. Another limitation that was identified was that none of the papers explored how pain clinics helped youth cope with their chronic pain. There is a need for additional qualitative studies grounded in youth’s experiences with a strict focus on youth coping with chronic pain, including further examination on the effectiveness of different coping methods, how youth coped with their pain based on their specific chronic pain conditions, and when they employed certain coping strategies. Such research will contribute to theory-building specific to youth living with chronic pain. Lastly, only papers written in English were included, limiting the findings by excluding papers published in other languages.

**Conclusion**

This systematic review aimed to synthesize insights from qualitative studies examining the experiences of youth with chronic pain and their coping strategies. From this, we arrived at two synthesized findings from 20 included studies. These synthesized findings highlighted the meanings youth assigned to living with their chronic pain and the complex experiences they have (*A Different Way of Being*), ad well as the self-directed strategies they employed and the external supports they relied on from family, friends, online communities, and professionals within the health care system (*Learning to Get By*). This highlighted that young people have unique and complicated experiences living with chronic pain, diverse methods of coping methods with their chronic pain, and challenging interactions with the health care system and seeking proper care. Future research should be undertaken to examine the coping experiences of racial minority youth with chronic pain, as well as male and gender diverse youth with chronic pain. Findings from this study can be used to support the well-being of youth living with chronic pain.

**Acknowledgements**

We thank Kristy Hancock, MLIS (Evidence Synthesis Coordinator, Maritime SPOR SUPPORT Unit) for peer review of the MEDLINE search strategy.

**Disclosure of Interest**

The authors declare that there are no conflicts of interest.

**Funding Details**

This study was supported by an operating grant from the Canadian Institutes of Health Research (Grant#: CIHR PJT-180639). RLW is supported by a is supported by a Tier 1 Canadian Research Chair in Child and Family Engagement in Health Research and Healthcare (Canadian Institutes of Health Research-Canadian Research Chair-950–231845).

**References**

1. Tutelman PR, Langley CL, Chambers CT, Parker JA, Finley GA, Chapman D, Jones GT, Macfarlane GJ, Marianayagam J. Epidemiology of chronic pain in children and adolescents: a protocol for a systematic review update. BMJ Open. 2021; 11(2): e043675. doi:10.1136/bmjopen-2020-043675

2. The Canadian Pain Task Force. Chronic pain in Canada: laying a foundation for action. 2019. https://www.canada.ca/en/health-canada/corporate/about-health-canada/public-engagement/external-advisory-bodies/canadian-pain-task-force/report-2019.html

3. Treede RD, Rief W, Barke A, Aziz Q, Bennett MI, Benoliel R, Cohen M, Evers S, Finnerup NB, First MB et al. A classification of chronic pain for ICD-11. Pain. 2015; 156(6): 1003-1007. doi:10.1097/j.pain.0000000000000160

4. World Health Organization. ICD-11 international classification of diseases for mortality and morbidty statistics. https://icd.who.int/browse/2024-01/mms/en

5. Palermo TM, Valrie CR, Karlson CW. Family and parent influences on pediatric chronic pain: a developmental perspective. Am Psychol. 2014; 69(2): 142.

6. Birnie KA, Killackey T, Stinson J, Noel M, Lorenzetti DL, Marianayagam J, Jordan I, Jordan E, Neville A, Pavlova M et al. Best practices for virtual care to support youth with chronic pain and their families: a rapid systematic review to inform health care and policy during COVID-19 and beyond. Pain Rep. 2021; 6(2): e935. doi:10.1097/PR9.0000000000000935

7. Noel M, Groenewald CB, Beals-Erickson SE, Gebert JT, Palermo TM. Chronic pain in adolescence and internalizing mental health disorders: a nationally representative study. Pain. 2016; 157(6): 1333-1338. doi:10.1097/j.pain.0000000000000522

8. Walker LS, Dengler-Crish CM, Rippel S, Bruehl S. Functional abdominal pain in childhood and adolescence increases risk for chronic pain in adulthood. Pain. 2010; 150(3): 568-572. doi:10.1016/j.pain.2010.06.018

9. Kashikar-Zuck S, Cunningham N, Peugh J, Black WR, Nelson S, Lynch-Jordan AM, Pfeiffer M, Tran ST, Ting TV, Arnold LM et al. Long-term outcomes of adolescents with juvenile-onset fibromyalgia into adulthood and impact of depressive symptoms on functioning over time. Pain. 2019; 160(2): 433-441. doi:10.1097/j.pain.0000000000001415

10. Breivik H, Collett B, Ventafridda V, Cohen R, Gallacher D. Survey of chronic pain in Europe: prevalence, impact on daily life, and treatment. Eur J Pain. 2006; 10(4): 287-333. doi:10.1016/j.ejpain.2005.06.009

11. Goldberg DS, McGee SJ. Pain as a global public health priority. BMC Public Health. 2011; 11(770).

12. Gureje O, Von Korff M, Kola L, Demyttenaere K, He Y, Posada-Villa J, Lepine JP, Angermeyer MC, Levinson D, de Girolamo G et al. The relation between multiple pains and mental disorders: results from the World Mental Health Surveys. Pain. 2008; 135(1-2): 82-91. doi:10.1016/j.pain.2007.05.005

13. Chambers CT, Dol J, Tutelman PR, Langley CL, Parker JA, Cormier BT, Macfarlane GJ, Jones GT, Chapman D, Proudfoot N et al. The prevalence of chronic pain in children and adolescents: a systematic review update and meta-analysis. Pain. 2024; 165(10): 2215-2234. doi:10.1097/j.pain.0000000000003267

14. King S, Chambers CT, Huguet A, MacNevin RC, McGrath PJ, Parker L, MacDonald AJ. The epidemiology of chronic pain in children and adolescents revisited: a systematic review. Pain. 2011; 152(12): 2729-2738. doi:10.1016/j.pain.2011.07.016

15. Huguet A, Miro J. The severity of chronic pediatric pain: an epidemiological study. J Pain. 2008; 9(3): 226-236. doi:10.1016/j.jpain.2007.10.015

16. Gobina I, Villberg J, Valimaa R, Tynjala J, Whitehead R, Cosma A, Brooks F, Cavallo F, Ng K, de Matos MG et al. Prevalence of self-reported chronic pain among adolescents: evidence from 42 countries and regions. Eur J Pain. 2019; 23(2): 316-326. doi:10.1002/ejp.1306

17. Stanford EA, Chambers CT, Biesanz JC, Chen E. The frequency, trajectories and predictors of adolescent recurrent pain: a population-based approach. Pain. 2008; 138(1): 11-21. doi:10.1016/j.pain.2007.10.032

18. Rabbitts JA, Holley AL, Groenewald CB, Palermo TM. Association between widespread pain scores and functional impairment and health-related quality of life in clinical samples of children. J Pain. 2016; 17(6): 678-684. doi:10.1016/j.jpain.2016.02.005

19. De Inocencio J. Epidemiology of musculoskeletal pain in primary care. Arch Dis Child. 2004; 89(5): 431-434. doi:10.1136/adc.2003.028860

20. Neville A, Jordan A, Beveridge JK, Pincus T, Noel M. Diagnostic uncertainty in youth with chronic pain and their parents. J Pain. 2019; 20(9): 1080-1090. doi:10.1016/j.jpain.2019.03.004

21. Alsaggaf F, Coyne I. A systematic review of the impact of chronic pain on adolescents' school functioning and school personnel responses to managing pain in the schools. J Adv Nurs. 2020; 76(8): 2005-2022.

22. Shelby GD, Shirkey KC, Sherman AL, Beck JE, Haman K, Shears AR, Horst SN, Smith CA, Garber J, Walker LS. Functional abdominal pain in childhood and long-term vulnerability to anxiety disorders. Pediatrics. 2013; 132(3): 475-482. doi:10.1542/peds.2012-2191

23. Walker LS, Sherman AL, Bruehl S, Garber J, Smith CA. Functional abdominal pain patient subtypes in childhood predict functional gastrointestinal disorders with chronic pain and psychiatric comorbidities in adolescence and adulthood. Pain. 2012; 153(9): 1798-1806. doi:10.1016/j.pain.2012.03.026

24. Leino-Arjas P, Rajaleid K, Mekuria G, Nummi T, Virtanen P, Hammarstrom A. Trajectories of musculoskeletal pain from adolescence to middle age: the role of early depressive symptoms, a 27-year follow-up of the Northern Swedish Cohort. Pain. 2018; 159(1): 67-74. doi:10.1097/j.pain.0000000000001065

25. Wrona SK, Melnyk BM, Hoying J. Chronic pain and mental health co-morbidity in adolescents: an urgent call for assessment and evidence-based intervention. Pain Manag Nurs. 2021; 22(3): 252-259. doi:10.1016/j.pmn.2020.12.004

26. Maes M, Van den Noortgate W, Fustolo-Gunnink SF, Rassart J, Luyckx K, Goossens L. Loneliness in children and adolescents with chronic physical conditions: a meta-analysis. J Pediatr Psychol. 2017; 42(6): 622-635. doi:10.1093/jpepsy/jsx046

27. Groenewald CB, Law EF, Fisher E, Beals-Erickson SE, Palermo TM. Associations between adolescent chronic pain and prescription opioid misuse in adulthood. J Pain. 2019; 20(1): 28-37. doi:10.1016/j.jpain.2018.07.007

28. Brattberg G. Do pain problems in young school children persist into early adulthood? A 13-year follow-up. Eur J Pain. 2004; 8(3): 187-199. doi:10.1016/j.ejpain.2003.08.001

29. Compas BE, Connor-Smith JK, Saltzman H, Thomsen AH, Wadsworth ME. Coping with stress during childhood and adolescence: problems, progress, and potential in theory and research. Psychol Bull. 2001; 127(1): 87-127. doi:10.1037/0033-2909.127.1.87

30. Carozza L, Anderson-Mackay E, Blackmore AM, Kirkman HA, Ou J, Smith N, Love S. Chronic pain in young people with cerebral palsy: activity limitations and coping strategies. Pediatr Phys Ther. 2022; 34(4): 489-495. doi:10.1097/PEP.0000000000000943

31. Yetwin AK, Mahrer NE, John C, Gold JI. Does pain intensity matter? The relation between coping and quality of life in pediatric patients with chronic pain. J Pediatr Nurs. 2018; 40: 7-13. doi:10.1016/j.pedn.2018.02.003

32. Lee S, Tomlinson R, Lumley MN, Bax KC, Ashok D, McMurtry CM. Positive schemas, coping, and quality of life in pediatric recurrent abdominal pain. J Clin Psychol Med Settings. 2024; 31(1): 37-47. doi:10.1007/s10880-023-09952-6

33. Murray CB, Groenewald CB, de la Vega R, Palermo TM. Long-term impact of adolescent chronic pain on young adult educational, vocational, and social outcomes. Pain. 2020; 161(2): 439-445. doi:10.1097/j.pain.0000000000001732

34. Compas BE, Jaser SS, Bettis AH, Watson KH, Gruhn MA, Dunbar JP, Williams E, Thigpen JC. Coping, emotion regulation, and psychopathology in childhood and adolescence: a meta-analysis and narrative review. Psychol Bull. 2017; 143(9): 939-991. doi:10.1037/bul0000110

35. Khanom S, McDonagh JE, Briggs M, Bakir E, McBeth J. Adolescents’ experiences of fluctuating pain in musculoskeletal disorders: a qualitative systematic review and thematic synthesis. BMC Musculoskelet Disord. 2020; 21(1): 1-16.

36. Nelson S, Agoston M, Kovar-Gough I, Cunningham N. A scoping review and proposed framework for coping in youth with a history of psychological trauma and chronic pain. J Pediatr Psychol. 2022; 7(4): 469-482.

37. Fegran L, Johannessen B, Ludvigsen MS, Westergren T, Hoie M, Slettebo A, Rohde G, Helseth S, Haraldstad K. Experiences of a non-clinical set of adolescents and young adults living with persistent pain: a qualitative metasynthesis. BMJ Open. 2021; 11(4): e043776. doi:10.1136/bmjopen-2020-043776

38. Tong A, Flemming K, McInnes E, Oliver S, Craig J. Enhancing transparency in reporting the synthesis of qualitative research: ENTREQ. BMC Med Res Methodol. 2012; 12(1): 181. doi:10.1186/1471-2288-12-181

39. McGowan J, Sampson M, Salzwedel DM, Cogo E, Foerster V, Lefebvre C. PRESS peer review of electronic search strategies: 2015 guideline statement. J Clin Epidemiol. 2016; 75: 40-46.

40. DeJean D, Giacomini M, Simeonov D, Smith A. Finding qualitative research evidence for health technology assessment. Qual Health Res. 2016; 26(10): 1307-1317. doi:10.1177/1049732316644429

41. CADTH Search Filters Database. Scopus NOT Medline/PubMed NOT Embase - Scopus. Ottawa: CADTH; 2022. https://searchfilters.cadth.ca/link/97

42. The EndNote Team. EndNote. Philadelphia, PA: Clarivate; 2013.

43. Veritas Health Innovation. Covidence systematic review software. Melbourne, Australia;

44. Neilson C. Search strategy for coping in young people living with chronic pain: a systematic review of qualitative evidence. University of Manitoba. 2023. doi:10.34990/FK2/7KMUD2

45. United Nations Youth. Definition of youth [fact sheet]. n.d. https://www.un.org/esa/socdev/documents/youth/fact-sheets/youth-definition.pdf

46. Backes EP, Bonnie RJ. The promise of adolescence: Realizing opportunity for all youth. Pain. 2019; 152(12): 2729-2738.

47. Reiss JG, Gibson RW, Walker LR. Health care transition: youth, family, and provider perspectives. Pediatrics. 2005; 115(1): 112-120.

48. Hong QN, Pluye P, Fabregues S, Bartlett G, Boardman F, Cargo M, Dagenais P, Gagnon MP, Griffiths F, Nicolau B et al. Mixed methods appraisal tool (MMAT) version 2018: user guide. McGill Department of Family Medicine; 2018.

49. Alsaggaf F, Coyne I. Participation in everyday life for young people with chronic pain in Saudi Arabia: “you feel lacking in life and you feel that time is flying by”. Front Rehabil Sci. 2023; 4: 1099345.

50. Carter B, Lambrenos K, Thursfield J. A pain workshop: an approach to eliciting the views of young people with chronic pain. J Clin Nurs. 2002; 11(6): 753-762.

51. Castle K, Imms C, Howie L. Being in pain: a phenomenological study of young people with cerebral palsy. Dev Med Child Neurol. 2007; 49(6): 445-449.

52. Forgeron PA, McGarth PJ. Self-identified needs of youth with chronic pain. J Pain Manag. 2008; 1(2): 163-172.

53. Forgeron PA, Evans J, McGarth PJ, Stevens B, Finley GA. Living with difference: exploring the social self of adolescents with chronic pain. Pain Res Manag. 2013; 18(6): 115-123.

54. Ghio D, Calam R, Lee RR, Cordingley L, Ulph F, Childhood Arthritis Prospective Study. "I just want to be normal": a qualitative investigation of adolescents' coping goals when dealing with pain related to arthritis and the underlying parent-adolescent personal models. Paediatr Neonatal Pain. 2022; 4(3): 96-109. doi:10.1002/pne2.12069

55. Gremillion ML, Lang AC, Everhart SA, Davies WH, Stolzman SC, Weisman SJ, Hainsworth KR. Effects of weight and pain on physical activity: insights from the lived experiences of youth with co-occurring chronic pain and obesity. Child Obes. 2022; 18(5): 301-308. doi:10.1089/chi.2021.0208

56. Hurley-Wallace A, Kirby S, Bishop F. Trusting in the online ‘community’: an interview study exploring internet use in young people with chronic pain. Br J Pain. 2022; 16(3): 341-353.

57. Jones A, Caes L, Eccleston C, Noel M, Gauntlett-Gilbert J, Jordan A. The sands of time: adolescents' temporal perceptions of peer relationships and autonomy in the context of living with chronic pain. Paediatr Neonatal Pain. 2022; 4(3): 110-124. doi:10.1002/pne2.12071

58. Killackey T, Soltani S, Noel M, Birnie KA, Choiniere M, Page MG, Dassieu L, Lacasse A, Lalloo C, Poulin P et al. "We survived the pandemic together": the impact of the COVID-19 pandemic on Canadian families living with chronic pain. Can J Pain. 2023; 7(2): 2157251. doi:10.1080/24740527.2022.2157251

59. Mahon PR, Reynolds D. Lived experiences of adolescents living with primary chronic pain. Pain Manag Nurs. 2024; 25(1): 19-26.

60. McKinnon CT, White JH, Morgan PE, Antolovich GC, Clancy CH, Fahey MC, Harvey AR. The lived experience of chronic pain and dyskinesia in children and adolescents with cerebral palsy. BMC Pediatr. 2020; 20(1): 125. doi:10.1186/s12887-020-2011-8

61. Meldrum ML, Tsao JC, Zelzer LK. "Just be in pain and just move on”: functioning limitations and strategies in the lives of children with chronic pain. J Pain Manag. 2008; 1(2): 131.

62. Nkhoma KB, Mwalabu GT, Bristowe K, Lungu EA, Harding R. Stakeholders' perspectives and requirements on pain self-management for adolescents living with HIV/AIDS in Malawi: a cross-sectional qualitative study. AIDS Care. 2023; 35(8): 1125-1138. doi:10.1080/09540121.2021.1939850

63. Parsons RD, McParland JL, Halligan SL, Goubert L, Jordan A. Glass half full: a diary and interview qualitative investigation of flourishing among adolescents living with chronic pain. Eur J Pain. 2023; 27(7): 896-911.

64. Root K, Nosek S. Understanding how college students characterise and cope with chronic pain: a thematic analysis of expressive writing samples. Med Humanit. 2023; 49(3): 370-377. doi:10.1136/medhum-2022-012466

65. Shaygan M, Jaberi A. The effect of a smartphone-based pain management application on pain intensity and quality of life in adolescents with chronic pain. Sci Rep. 2021; 11(1): 6588. doi:10.1038/s41598-021-86156-8

66. Skogvold L, Magnussen LH. Chronic tension-type headache and coping strategies in adolescents: a qualitative interview study. Physiother Res Int. 2019; 24(3): e1778. doi:10.1002/pri.1778

67. Stinson JN, Lalloo C, Harris L, Issac L, Campbell F, Brown S, Karim A. iCanCope with Pain™: user-centred design of a web-and mobile-based self-management program for youth with chronic pain based on identified health care needs. Pain Res Manag. 2014; 19(5): 257-265.

68. Szwimer R, Widjaja M, Ingelmo P, Hovey RB. A phenomenological exploration of the personal implications of female adolescents living with chronic pain. J Pediatr Health Care. 2020; 34(5): 470-477.

69. Thomas J, Harden A. Methods for the thematic synthesis of qualitative research in systematic reviews. BMC Med Res Methodol. 2008; 8: 45. doi:10.1186/1471-2288-8-45

70. Patton M. Qualitative research & evaluation methods: integrating theory and practice. 4th ed: Sage; 2014.

71. Barbour R. Introducing qualitative research: a student guide to the craft of doing qualitative research. Sage; 2008.

72. Morse J, Field P. Qualitative research methods for health professionals. Sage; 1995.

73. Rosenbloom BN, Rabbitts JA, Palermo TM. A developmental perspective on the impact of chronic pain in late adolescence and early adulthood: Implications for assessment and intervention. Pain. 2017; 158(9): 1629-1632. doi:10.1097/j.pain.0000000000000888

74. Barberis N, Gugliandolo MC, Costa S, Liga F. How parental autonomy support prevent from adolescents’ depression and low self-esteem: a mediational model with trait emotional intelligence. Mediterr J Clin Psychol. 2021; 9(1): 1-18. doi:10.6092/2282-1619/mjcp-2898

75. Jaworski N, MacQueen G. Adolescence as a unique developmental period. J Psychiatry Neurosci. 2015; 40(5): 291-293. doi:10.1503/jpn.150268

76. Twiddy H, Hanna J, Haynes L. Growing pains: understanding the needs of emerging adults with chronic pain. Br J Pain. 2017; 11(3): 108-118.

77. Stinson J, Connelly M, Kamper SJ, Herlin T, Toupin AK. Models of care for addressing chronic musculoskeletal pain and health in children and adolescents. Best Pract Res: Clin Rheumatol. 2016; 30(3): 468-482. doi:10.1016/j.berh.2016.08.005

78. Linkiewich D, Dib KC, Forgeron PA, Dick BD, McMurtry CM. Perceptions of adolescents with chronic pain about peer support: reflexive thematic analysis. J Pediatr Psychol. 2023; 48(8): 655-663. doi:10.1093/jpepsy/jsad014

79. Newton BJ, Southall JL, Raphael JH, Ashford RL, Lemarchand K. A narrative review of the impact of disbelief in chronic pain. Pain Manag Nurs. 2013; 14(3): 161-171. doi:10.1016/j.pmn.2010.09.001

80. Meldrum ML, Tsao JCI, Zeltzer LK. “I can't be what I want to be”: children's narratives of chronic pain experiences and treatment outcomes. Pain Med. 2009; 10(6): 1018-1034. doi:10.1111/j.1526-4637.2009.00650.x

81. Wakefield EO, Zempsky WT, Puhl RM, Litt MD. Conceptualizing pain-related stigma in adolescent chronic pain: A literature review and preliminary focus group findings. Pain Rep. 2018; 3: e679. doi:10.1097/PR9.0000000000000679

82. Health Standards Organization. Pediatric pain management. 2023. https://healthstandards.org/standard/pediatric-pain-management-can-hso-13200-2023-e/

83. Institute of Medicine. Relieving pain in America: a blueprint for transforming prevention, care, education, and research. Washington, DC: The National Academies Press; 2011.

84. Gatchel RJ, Peng YB, Peters ML, Fuchs PN, Turk DC. The biopsychosocial approach to chronic pain: scientific advances and future directions. Psychol Bull. 2007; 133(4): 581-624. doi:10.1037/0033-2909.133.4.581

85. Collins J, Haynes N, Klingberg H, Nicholas H, Pounder M, Sandells R. The management of complex pain in children referred to a pain clinic at a tertiary children’s hospital in Australia. J Orthop Sports Phys Ther. 2017; 47(10): 806-813. doi:10.2519/jospt.2017.7355

86. Harrison LE, Pate JW, Richardson PA, Ickmans K, Wicksell RK, Simons LE. Best-evidence for the rehabilitation of chronic pain part 1: pediatric pain. J Clin Med. 2019; 8(9): 1267. doi:10.3390/jcm8091267

87. Choinière M, Peng P, Gilron I, Buckley N, Williamson O, Janelle-Montcalm A, Baerg K, Boulanger A, Di Renna T, Finley GA et al. Accessing care in multidisciplinary pain treatment facilities continues to be a challenge in Canada. Reg Anesth Pain Med. 2020; 45(12): 943-948. doi:10.1136/rapm-2020-101935

88. Wittmeier K, Brown C, Diaz F, Pylypjuk H, Restall G, Anang P, Gerhold K. Collaborating with a youth council to improve chronic pain resources. Canadian Journal of Pain. 2023; 7(1): 2254358. doi:10.1080/24740527.2023.2254358
